# Elevated inflammation supra-additively promotes the progression from prediabetes to diabetes: a prospective cohort study

**DOI:** 10.7189/jogh.15.04318

**Published:** 2025-10-24

**Authors:** Yulong Lan, Dan Wu, Huancong Zheng, Xiong Ding, Hui Zhou, Kuangyi Wu, Weiqiang Wu, Zegui Huang, Xianxuan Wang, Wei Wang, Shouling Wu, Youren Chen

**Affiliations:** 1 Second Affiliated Hospital of Shantou University Medical College, Department of Cardiology, Shantou, China; 2 Edith Cowan University, School of Medical and Health Sciences, Centre for Precision Health, Joondalup, Australia; 3 Wuhan University, School of Public Health, Wuhan, China; 4 Central South University, Xiangya School of Nursing, Changsha, China; 5 University of Toronto, Department of Physiology, Temerty Faculty of Medicine, Toronto, Canada; 6 Capital Medical University, Beijing Key Laboratory of Clinical Epidemiology, School of Public Health, Beijing, China; 7 Shandong First Medical University & Shandong Academy of Medical Sciences, School of Public Health, Tai'an, China; 8 Kailuan General Hospital, Department of Cardiology, Tangshan, China

## Abstract

**Background:**

Factors impacting on the conversion of prediabetes to diabetes or normoglycemia remain unclear. This study aimed to investigate the role of subclinical inflammation, assessed by high-sensitivity C-reactive protein (hsCRP), in the progression to diabetes from prediabetes, assessed by impaired fasting glucose (IFG).

**Methods:**

Time-to-event survival analyses were conducted among 82 475 participants without diabetes from Kailuan Study (a real-life prospective cohort in China) to access the isolated and joint effect of hsCRP and IFG on diabetes risk, and quantify their relative contribution to incident diabetes.

**Results:**

Over a median 11-year follow-up, 14 215 diabetes cases were recorded. IFG and hsCRP independently and jointly increased diabetes risk. Diabetes incidence was higher in those with elevated inflammation (hsCRP≥2 mg/L: 90.45 *vs*. 66.76 per 1000 person-years). The joint effect risk (hazard ratios (HR) = 4.96; 95% confidence interval (CI) = 4.66–5.28) exceeded the sum of individual risks (HR = 4.29; 95% CI = 4.09–4.49 for IFG and HR = 1.11; 95% CI = 1.06–1.16 for elevated inflammation), with a relative excess risk due to interaction of 0.56 (95% CI = 0.23–0.89). Attributable proportions were 83.08% for IFG, 2.78% for hsCRP, and 14.14% for their interaction. The joint risks and the additive interaction were significant in both men and women, and were more pronounced among individuals aged <60 years than those aged ≥60 years.

**Conclusions:**

Elevated inflammation synergistically amplifies diabetes risk in prediabetes among Chinese adults, particularly in those <60 years.

Type 2 diabetes has become a scourge of our era, affecting over 451 million people globally in 2017 [1]. Prediabetes (also known as intermediate hyperglycemia) is a high-risk state that defines an elevated chance of developing diabetes [2]. The prevalence of prediabetes is increasing worldwide and affects one in three adults in the US and approximately 720 million individuals worldwide [1]. In mainland China, the estimated standardised prevalence was 10.9% for diabetes and 35.7% for prediabetes [3].

The reported annualised conversion rate from prediabetes to diabetes was 5–10%, with a similar proportion converting back to normoglycemia [4]. Although the reasons for this heterogeneity are incompletely understood, elevated inflammation has been proposed to play a key role in the progression of prediabetes to diabetes [5,6]. Genetic and protein levels of pro-inflammatory markers were significantly elevated in prediabetes [7]. For example, data were accumulated for the increased levels of high-sensitivity C-reactive protein (hsCRP) [8,9] and interleukin-1 (IL) [10] in prediabetes. A sustained inflammatory response may contribute to β-cell dysfunction that fails to compensate for the insulin need in the context of insulin resistance (IR), thereby promoting prediabetes to diabetes onset [6]. A laboratory-based study on mouse models suggested that chronic low-grade inflammation produces circulating cytokine levels that are sufficient to induce beta-cell dysfunction and plays a pathological role in beta-cell failure in early type 2 diabetes [11]. The results of the Hong Kong Cardiovascular Risk Factors Prevalence Study suggested that prediabetic individuals with elevated CRP levels had an approximately 3-fold risk of remaining in prediabetes or progressing to diabetes in a 2-year period [12]. Moreover, preclinical studies that translate the potential of diabetes prevention with anti-inflammatory agents to practice have been conducted in recent decades [13–17]. Despite limitations of small sample size and short-term duration that may confound the elevation of the real effect of anti-inflammation on preventing diabetes, the consistent reduction in overall inflammation during the treatment and overall positive effect on improving β-cell biology and glucose hemostasis was suggested [18,19]. A recent meta-analysis demonstrated a significant benefit of IL-1 antagonists against diabetes [20].

Although anti-inflammatory treatment has been considered an important supplement to lifestyle modification for diabetes prevention, evidence on the relative contribution of elevated inflammation to the progression of prediabetes to type 2 diabetes is sparse. Therefore, we aimed to provide population-based epidemiological evidence regarding the effect of elevated inflammation on the development of type 2 diabetes. A specific investigation on the potential biological interaction of subclinical inflammation and prediabetic status and their relative contribution to diabetogenesis among 82 754 individuals without preexisting diabetes from a real-life, prospective cohort (the Kailuan Study) was conducted.

## METHODS

### Study participants

The Kailuan Study (trial registration number: ChiCTR-TNC-11001489) has been previously described [21]. It is an ongoing, real-world, prospective cohort study conducted in Tangshan, Northern China and was approved by the Kailuan General Hospital Ethics Committee, China (2006-05). Baseline and follow-up health surveys (including standard questionnaires, anthropometrics, and biological tests) have been conducted every two years since 2006/2007. For the current analysis, among 101 510 participants who attended the baseline health visit (2006 / 2007), we excluded those with missing or extreme values for age, sex, weight, height, fasting blood glucose (FBG) and hsCRP levels (n = 3668) and those with known diabetes at the start of follow-up (n = 8518) as well as missing follow-up visits (n = 6849). Consequently, a total of 82 475 patients were included in the analysis. Figure S1 in the **Online Supplementary Document** displays the flowchart of participant enrollment. Characteristics of the included and excluded individuals are presented in Table S1 in the **Online Supplementary Document**. Each participant provided written informed consent before enrollment.

### Assessment of outcome

The primary outcome was type 2 diabetes (ICD-10 = E11) incidence. Type 2 diabetes was defined as either an FBG level of 7.0 mmol/L or greater, self-reported history of type 2 diabetes diagnosed by a physician, or self-reported use of oral antihyperglycemic agents with or without the use of insulin [22]. Person-time for each participant was counted from the return of the baseline questionnaire to the occurrence of the study outcome (the midpoint between the exact date of the visit at which incident diabetes was ascertained and the exact date of the previous visit), death, or last return of a valid follow-up survey prior to 31 December 2020.

### Exposures

Prediabetes was assessed by impaired fasting glucose (IFG), defined as any participants who did not have diabetes but who had FBG levels of 6.1 to 6.9 mmol/L according to World Health Organization (WHO) diagnostic criteria [23] or 5.6 to 6.9 mmol/L according to American Diabetes Association (ADA) criteria [24]. Body mass index (BMI) was calculated as weight in kilograms divided by height in meters squared. Inflammation was primarily assessed by hsCRP using the clinically established cutoff of ≥2 mg/L [16,25,26], with ≥3 mg/L [27,28] considered as a secondary threshold according to existing literature.

### Covariates

Baseline information was obtained through the health examination in 2006 / 2007. Multiple variables, including socio-demographics, anthropometrics, lifestyle data, medicine history, medication use (anti-hypertensives, lipid-lowering drugs), and biochemistry parameters, were collected [21]. The estimated glomerular filtration rate (eGFR) was calculated from creatinine for assessing renal function. Smoking and drinking status were defined according to average alcohol consumption in the past year, as detailed previously [29]. Smoking habits were categorised as never, former, or current smoker. Alcohol consumption was divided into ‘yes or no’.

### Statistical analysis

Baseline descriptive statistics are represented as the mean ± standard deviation (SD), median with interquartile range (IQR), or number and percentage (%), as appropriate. Missing values (Table S2 in the **Online Supplementary Document**) were imputed by chained multiple imputation under the missing-at-random assumption. Differences in baseline characteristics between prediabetes cases and non-cases were compared using the χ^2^ test for categorical variables and an unpaired Student’s *t* test or Mann-Whitney U test for continuous variables.

The proportional hazard assumption was assessed by visual inspection of the survival curves and the Kolmogorov-Smirnov test. Cox proportional hazard regression models were used to compare the risk (unadjusted and adjusted hazard ratios (aHRs) with 95% confidence intervals (CIs)) of type 2 diabetes onset with exposure to isolated and combined IFG and hsCRP levels. The diabetes incidence rates (per 1000 person-years) were calculated. Kaplan-Meier plots were generated with the log-rank test to compare the cumulative incidence across subgroups. We constructed a series of progressively adjusted Cox proportional hazards models. Model 1 was adjusted for age, sex, smoking habits, alcohol consumption, physical activity, education level, family history of diabetes, and lipid-lowering and anti-hypertensive drug use; Model 2 additionally included baseline blood pressure (continuous), renal function (continuous) and log (triglyceride (TG)/high-density lipoprotein cholesterol (HDL-C)) (continuous), log (hsCRP) (continuous, for isolated IFG exposure). Model 3 further adjusted for BMI (continuous) and Model 4 alternatively adjusted for waist circumference (continuous). The inclusion of BMI and waist circumference in separate models follows established practice in diabetes epidemiology [30–32] to distinguish the effects of overall and central adiposity. Thereafter, the diabetes risks were also obtained for IFG and hsCRP categories combined. The multiplicative interaction (INTm) between IFG and hsCRP levels was assessed by the likelihood-ratio test by including a cross-product term. The relative excess risk due to interaction (RERI) and attributable proportion due to interaction (AP) as indexes of supra-additive interaction [33] were calculated by using the HRs derived from our multivariable-adjusted Cox model, with non-IFG and non-elevated inflammation as the baseline. Briefly, on the hazard ratio scale, we decomposed the joint excess relative risk for both exposures (HR11-1) into the excess relative risk for elevated inflammation (HR01-1), IFG (HR10-1), and relative excess risk due to their interaction (RERI). Specifically, we have HR11-1 = (HR01-1) + (HR10-1) + RERI [34]. We then likewise calculated the proportion of the joint effect due to elevated inflammation, (HR01-1)/(HR11-1); due to IFG, (HR10-1)/(HR11-1) and due to their additive interaction, RERI/(HR11-1).

Additionally, we applied restricted cubic spline (RCS) models with three knots (at the 10th, 50th, and 90th percentiles) to examine potential dose-response relationships between log-transformed hsCRP and incident diabetes in the overall population and within IFG strata. Based on the spline pattern, we conducted an exploratory analysis using hsCRP>1 mg/L as an alternative cutoff. Sensitivity analyses were further performed to assess the robustness of the findings by excluding those with baseline CVD or those with suspected infection (hsCRP levels ≥10 mg/L) or on raw data. Evidence for age and sex heterogeneity across combined exposure was tested by means of a likelihood ratio test by adding multiplicative interaction terms to a proportional hazards model. Subgroup and interaction analyses were considered exploratory and not adjusted for multiple comparisons.

All *P*-values presented were two-sided, with statistical significance determined by a false discovery rate of less than 0.05. For the additive interaction, RERI and AP greater than zero were defined as a positive deviation and considered significant when the 95% CI did not contain zero. Unless otherwise stated, statistical analyses in this study were performed using SAS version 9.4 (SAS Institute, Cary, NC, USA).

## RESULTS

**Table 1** shows the baseline characteristics of the included 82 475 study participants – 66 257 (80.3%) males, mean (SD) age = 50.4 ± 12.0 years and according to categories of IFG cases and non-cases defined with WHO criteria. The proportion of elevated inflammation was 29.2% among prediabetic individuals *vs*. 25.6% among non-prediabetic participants. Compared to the non-cases, the prediabetes cases had higher BMI, waist circumference, blood pressure, total cholesterol, triglycerides and hsCRP levels as well as a greater prevalence of overweight/obesity, hypertension and dyslipidemia. Additionally, the IFG individuals tended to be current drinkers, ever and current smokers, physically inactive and had lower educational attainment.

**Table 1:** Baseline characteristics of the study participants

Variables	Total (n = 82 475)	IFG (WHO criteria) (n = 7353)	Non-IFG (WHO criteria) (n = 75 122)	*P-*difference
Age, mean (SD), years	50.4 ± 12.0	52.4 ± 10.7	50.2 ± 12.1	<0.0001
Male, No. (%)	66257 (80.3)	6391 (86.9)	59866 (79.7)	<0.0001
BMI, mean (SD), kg/m^2^	25.0 ± 12.0	25.9 ± 3.4	24.9 ± 3.4	<0.0001
Waist circumference, mean (SD), cm	86.6 ± 9.7	88.6 ± 9.6	86.4 ± 9.7	<0.0001
hsCRP, median (IQR), mg/L	0.8 (0.3–2.1)	0. 9 (0.4–2.4)	0.7 (0.3–2.0)	0.0094
hsCRP-categories, No. (%)				<0.0001
*<2 mg/L*	61060 (74.0)	5209 (70.8)	55851 (74.4)	
*≥2 mg/L*	21415 (26.0)	2144 (29.2)	19271 (25.6)	
SBP, mean (SD), mm Hg	129.7 ± 20.4	135.7 ± 21.0	129.1 ± 20.3	<0.0001
DBP, median (IQR), mm Hg	80.0 (78.0–90.0)	83.3 (80.0–91.3)	80.0 (76.7–90.0)	<0.0001
HDL-C, median (IQR), mmol/L	1.50 (1.28–1.76)	1.5 (1.3–1.8)	1.5 (1.3–1.8)	0.8957
LDL-C, mean (SD), mmol/LA	2.3 ± 0.90	2.5 ± 0.9	2.3 ± 0.9	<0.0001
TC, mean (SD), mmol/L	4.9 ± 1.1	5.1 ± 1.2	4.9 ± 1.1	<0.0001
TG, median (IQR), mmol/L	1.3 (0.8–1.9)	1.5 (1.0–2.3)	1.2 (0.9–1.8)	<0.0001
eGFR, median (IQR), ml/min/1.73m^2^	82.1 (68.9–96.6)	83.0 (69.0–98.9)	82.1 (68.1–96.4)	0.0020
Family history of diabetes, No. (%)	6842 (8.3)	731 (9.9)	6111 (8.1)	<0.0001
Education, No. (%)				<0.0001
*Less than high school*	65207 (79.1)	6040 (82.1)	59167 (78.8)	
*High school and above*	17268 (20.9)	1313 (17.9)	15955 (21.2)	
Current drinker	31373 (38.0)	3091 (42.0)	28282 (37.7)	<0.0001
Smoking habits, No. (%)				<0.0001
*Never smoker*	49281 (59.8)	4101 (55.8)	45180 (60.1)	
*Ever smoker*	4477 (5.4)	482 (6.5)	3995 (5.3)	
*Current smoker*	28717 (34.8)	2770 (37.7)	25947 (34.6)	
Physical activities, No. (%)				<0.0001
*Low*	7514 (9.1)	813 (11.1)	6701 (8.9)	
*Moderate*	62309 (75.6)	5295 (72.0)	57014 (75.9)	
*High*	12652 (15.3)	1245 (16.9)	11407 (15.2)	
CVD, No. (%)	2467 (3.0)	2220 (3.0)	247 (3.4)	0.0523
Hypertension, No. (%)	34087 (41.3)	3997 (54.4)	30090 (40.1)	<0.0001
Dyslipidaemia, No. (%)	24080 (29.2)	2790 (37.9)	21290 (28.3)	<0.0001
Medication use, No. (%)				
*Antihypertensives*	8303 (10.1)	1038 (14.1)	7265 (9.7)	<0.0001
*Statin*	176 (0.2)	19 (0.3)	157 (0.2)	0.3809
*Fibrate*	63 (0.08)	9 (0.12)	54 (0.07)	0.1346

BMI – body mass index, DBP – diastolic blood pressure, eGFR – estimated glomerular filtration rate, HDL-C – high-density lipoprotein cholesterol, hsCRP – high-sensitivity C-reactive protein, IFG – impaired fasting glucose, IQR – interquartile range, LDL-C – low-density lipoprotein cholesterol, SBP – systolic blood pressure, SD – standard deviation, TC – total cholesterol, TG – triglyceride, WHO – World Health Organization

**Figure 1:**
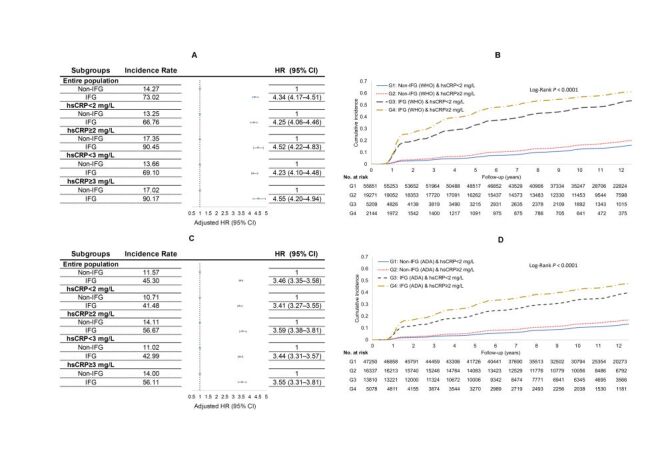
Risk of incident type 2 diabetes upon prediabetic status in the entire cohort and across levels of high-sensitivity C-reactive protein (hsCRP) and their joint exposure. **Panel A.** Associations between IFG statuses (WHO criteria) and incident type 2 diabetes in the entire population and across different hsCRP levels. **Panel B.** Cumulative incidence of type 2 diabetes with co-exposure to IFG (WHO criteria) and elevated inflammation (hsCRP≥2 mg/L). **Panel C.** Associations between IFG statuses (ADA criteria) and incident type 2 diabetes in the entire population and across different hsCRP levels. **Panel D.** Cumulative incidence of type 2 diabetes with co-exposure to IFG (ADA criteria) and elevated inflammation (hsCRP≥2 mg/L). For the association analysis, all models were adjusted for age (continuous), sex, family history of diabetes, medication use of antihypertensives and lipid-lowering drugs, education, physical activity, smoking habits, alcohol consumption, log(TG/HDL-C) (continuous), blood pressure categories (non-hypertension, hypertension grade I, grade II, grade III), eGFR (≥90, 60–90, 30–60, <30 ml/min/1.73m^2^), BMI (continuous), and log(hsCRP) (continuous, limited to the entire cohort).

A total of 14 215 participants developed type 2 diabetes during a median of 10.9 (interquartile range (IQR) = 6.9–12.6) years of follow-up. There was a positive association between grade increase in hsCRP levels or IFG status and type 2 diabetes incidence (**Figure 1**, Panels A–D; Table S3–6 in the **Online Supplementary Document**). Compared to hsCRP levels <2 mg/L, hsCRP≥2 mg/L was associated with a 21% increase in the risk of diabetes (HR = 1.21; 95% CI = 1.16–1.25) after adjusting for age, sex, lifestyle factors, family history of diabetes, blood pressure, renal function and log-normalised (TG/HDL-C) levels; per SD increase in log(hsCRP) was associated with a risk of 1.12 (95% CI = 1.10–1.14). The hsCRP-diabetes association was attenuated but remained statistically significant after additional adjustment for BMI or waist circumference (HR = 1.12; 95% CI = 1.08–1.16 for hsCRP≥2 *vs*. <2 mg/L). After adjusting for the mentioned sociodemographic and lifestyle factors and clinical information, IFG was significantly associated with incident diabetes compared to non-IFG (HR = 4.48; 95% CI = 4.31–4.66). The risk of diabetes was slightly attenuated by additional adjustment for BMI (HR = 4.34; 95% CI = 4.17–4.51) but not for waist circumference. When we examined the association of joint categories of IFG status and inflammation levels, within each inflammation level, IFG was consistently associated with a higher risk and incidence of type 2 diabetes. Higher IFG-associated type 2 diabetes incidences (90.45 *vs*. 66.76 per 1000 person-years for hsCRP≥2 *vs*. <2 mg/L) and risks (HR = 4.52; 95% CI = 4.22–4.83 *vs*. HR = 4.25; 95% CI = 4.06–4.46 for hsCRP≥2 *vs*. <2 mg/L) were observed in those with elevated inflammation. This trend remained when further adjusting for waist circumference instead of BMI. Tests for multiplicative interactions were not significant (*P* = 0.3451). Nonetheless, there was a significant additive interaction between IFG and elevated inflammation. In the multivariable adjusted model, the HRs were 4.29 (95% CI = 4.09–4.49) for IFG, 1.11 (95% CI = 1.06–1.16) for elevated inflammation, and 4.96 (95% CI = 4.66–5.28) for their joint effect, with a relative excess risk due to interaction of 0.56 (95% CI = 0.23–0.89). The attributable proportions of the joint effect were 83.08% for IFG alone, 2.78% for elevated inflammation alone, and 14.14% for their interaction (**Table 2**). The cumulative incidence and the incidence rates per 1000 years were much higher upon exposure to both elevated inflammation and IFG. When stratifying hsCRP levels as <1, 1 to 3, and ≥3 mg/L, there were positive graded associations between hsCRP strata and incident type 2 diabetes in the fully adjusted model. Across all levels of hsCRP, we documented a consistent and graded increasing risk of type 2 diabetes with IFG. Similar results were obtained when redefining elevated inflammation as hsCRP levels ≥3 mg/L (**Figure 1****,** Panel A and Panel C; Table S7–12 in the **Online Supplementary Document**).

**Table 2:** Additive effect of IFG and elevated inflammation on type 2 diabetes risks

Main effects – hazard ratios	Model 1*	Model 2*	Model 3*	Model 4*	
**According to WHO criteria, HR (CI)**				
IFG (WHO criteria)†	4.80 (4.59–5.03)	4.44 (4.24–4.65)	4.29 (4.09–4.49)	4.45 (4.25–4.66)	
hsCRP≥2 mg/L†	1.24 (1.19–1.30)	1.20 (1.15–1.25)	1.11 (1.06–1.16)	1.11 (1.06–1.16)	
Joint effect†	6.20 (5.83–6.59)	5.53 (5.18–5.87)	4.96 (4.66–5.28)	5.06 (4.75–5.38)	
RERI†	1.15 (0.75–1.56)	0.89 (0.52–1.25)	0.56 (0.23–0.89)	0.50 (0.16–0.84)	
AP†	0.19 (0.13–0.24)	0.16 (0.10–0.22)	0.11 (0.05–0.18)	0.10 (0.04–0.16)	
S†	1.29 (1.18–1.40)	1.24 (1.14–1.36)	1.17 (1.07–1.27)	1.14 (1.04–1.24)	
**Attributable proportion, %**				
IFG (WHO criteria)	73.07	75.93	83.08	84.98	
hsCRP≥2 mg/L	4.61	4.42	2.78	2.71	
Joint effect	22.12	19.65	14.14	12.31	
**According to ADA criteria, HR (CI)**				
IFG (ADA criteria)†	3.76 (3.61–3.92)	3.54 (3.40–3.69)	3.42 (3.29–3.56)	3.53 (3.39–3.67)	
hsCRP≥2 mg/L†	1.24 (1.18–1.31)	1.20 (1.14–1.26)	1.12 (1.06–1.17)	1.11 (1.06–1.17)	
Joint effect†	4.88 (4.63–5.13)	4.40 (4.18–4.64)	3.97 (3.77–4.18)	4.07 (3.86–4.28)	
RERI†	0.88 (0.63–1.11)	0.66 (0.44–0.88)	0.43 (0.23–0.63)	0.43 (0.22–0.64)	
AP†	0.18 (0.14–0.22)	0.15 (0.11–0.20)	0.11 (0.06–0.16)	0.11 (0.06–0.15)	
S†	1.29 (1.21–1.38)	1.24 (1.16–1.33)	1.17 (1.09–1.26)	1.16 (1.08–1.25)	
**Attributable proportion, %**				
IFG (ADA criteria)	71.13	74.71	81.48	82.41	
hsCRP≥2 mg/L	6.19	5.88	4.04	3.58	
Joint effect	22.68	19.41	14.48	14.01	

ADA – American Diabetes Association, AP – attributable proportion due to interaction, CI – confidence interval, HR – hazard ratios, hsCRP – high-sensitivity C-reactive protein, IFG – impaired fasting glucose, RERI – relative excess risk due to interaction, S – synergy index, WHO – World Health Organization

*Model 1: adjusted for sex, age, smoking habits, alcohol consumption, physical activities, education, family history of diabetes, antihypertensives, and lipid-lowering drugs. Model 2: further adjusted for *log* (TG/HDL-C) (continuous), blood pressure categories (non-hypertension, hypertension grade I, grade II, grade III), and eGFR (≥90, 60-90, 30-60, <30 ml/min/1.73m^2^). Model 3: Model 2 + BMI (continuous). Model 4: Model 2 + waist circumference (continuous).

†Baseline category was defined as non-IFG and hsCRP<2 mg/L.

In the RCS models, log-transformed hsCRP showed a generally positive association with diabetes risk across the overall population and IFG subgroups. Among individuals with prediabetes, the curve indicated an increased risk above hsCRP ≈ 1 mg/L, with the slope gradually flattening thereafter (Figure S2 in the **Online Supplementary Document**). Using hsCRP>1 mg/L as an alternative cutoff yielded similar risk of developing type 2 diabetes and supra-additive interactions with IFG (Table S13–14 in the **Online Supplementary Document**).

When defining IFG in accordance with the ADA criteria, IFG was consistently associated with higher type 2 diabetes incidence and risks. Despite a nonsignificant multiplicative interaction, individuals with elevated inflammation tended to have higher IFG-associated absolute risk (56.67 *vs*. 41.48 per 1000 person-years for hsCRP≥2 *vs*. <2 mg/L) and adjusted risks (3.59; 95% CI = 3.38–3.81 *vs*. 3.41; 95% CI = 3.27–3.55 for hsCRP≥2 *vs*. <2 mg/L) for incident type 2 diabetes. Compared with those having no exposure to IFG or elevated inflammation, the aHRs were 1.12 (95% CI = 1.06–1.17) for exposure to simply elevated inflammation, 3.42 (95% CI = 3.29–3.56) for exposure to simply IFG, and 3.97 (95% CI = 3.77–4.18) for exposure to both elevated inflammation and IFG, with an RERI of 0.43 (95% CI = 0.23–0.63). The attributable proportions of the joint effect were 81.48% for IFG alone, 4.04% for elevated inflammation alone, and 14.48% for their interaction. Similar results were obtained when redefining elevated inflammation as hsCRP levels ≥3 mg/L (Table S15–20 in the **Online Supplementary Document**).

**Figure 2:**
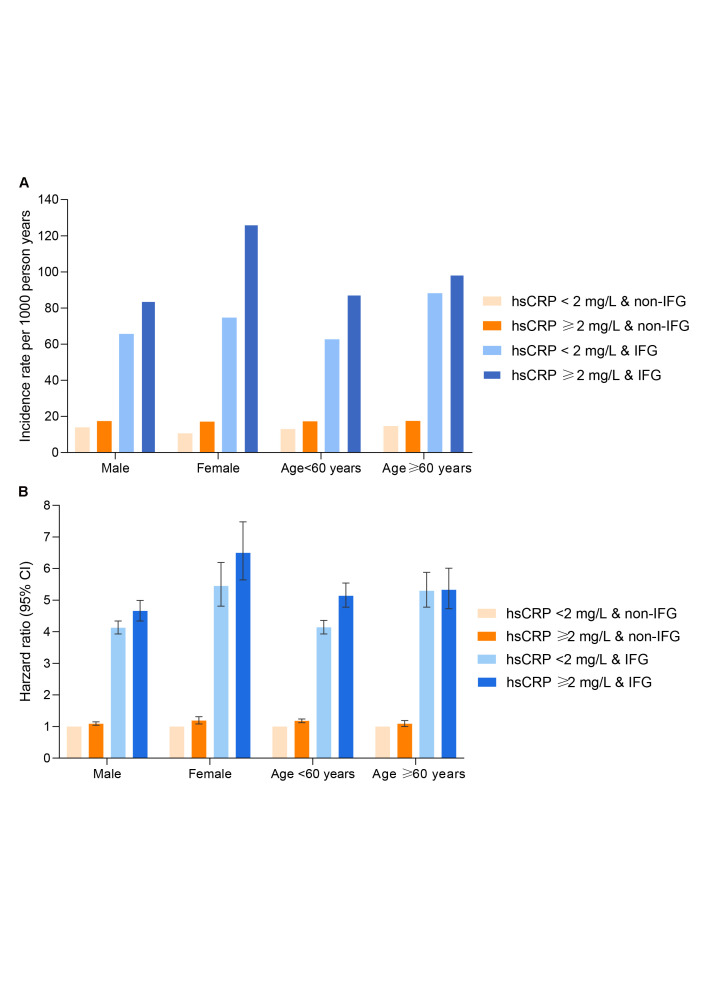
Sex, age-stratified analysis of the risks of diabetes upon coexposure to prediabetes and elevated inflammation. **Panel A.** Unadjusted type 2 diabetes incidence per 1000 person years across sex (male, female) and age (<60, ≥60 years) subgroups. **Panel B.** Multivariable-adjusted hazard ratios (95% CI) of incident type 2 diabetes across sex (male, female) and age (<60, ≥60 years) subgroups; all models were adjusted for age, sex, education, smoking status, drinking status, physical activity, family history of diabetes, medication use of anti-hypertensives and lipid-lowering drugs, *log*(TG/HDL-C) (continuous), blood pressure categories (non-hypertension, hypertension grade I, grade II, grade III), eGFR (≥90, 60-90, 30-60, <30 ml/min/1.73m^2^) and BMI (continuous).

The results remained similar to the primary results after excluding individuals with preexisting CVD, with suspected infection, or with incomplete data on study covariates (Table S21–24 in the **Online Supplementary Document**). There were significant risk heterogeneities across age and sex subgroups (both *P*-INTm <0.0001). Females exhibited higher diabetes incidences and risks with co-exposure to elevated inflammation and IFG compared to males. Notably, measures of additive interaction were statistically significant in both men and women, but were evident only among participants younger than 60 years and not in those aged ≥60 years (**Figure 2**, Panel A–B; Table S25–28 in the **Online Supplementary Document**).

## DISCUSSION

In a real-life, prospectively designed cohort in China, elevated inflammation and prediabetes were independently and jointly associated with a higher risk of incident type 2 diabetes. Elevated inflammation synergistically enhanced the risks of incident diabetes in prediabetes, with a supra-additive interaction between elevated inflammation and prediabetes found, independent of body weight (BMI or waist circumference). The proportions of the joint association were 81.48% for prediabetes (WHO criteria) alone, 4.04% for elevated inflammation alone, and 14.48% for an additive interaction between elevated inflammation and prediabetes. We observed consistent supra-additive interactions in both sexes, whereas the age-specific analyses revealed that such effects were restricted to participants younger than 60 years.

Prediabetes identified as having either or both impaired fasting glucose and impaired glucose tolerance predicts increased risks of incident diabetes in many populations. Our findings highlight the significant supra-additive interaction between prediabetes and elevated inflammation on the risk of type 2 diabetes, independent of body weight. If both elevated inflammation and prediabetes were present, this would result in an additional 14.48% of type 2 diabetes cases. Elevated inflammation and islet dysfunction were pronounced in people with prediabetes [7,9,10]. Compelling evidence from well-designed cohorts and clinical trials has accumulated for a proven pathological role of elevated inflammation in diabetogenesis that elevated inflammation significantly contributes to a higher risk of progressing to diabetes from prediabetes [12] and that pharmacologic anti-inflammatory treatment improves glycemic metabolism [13–17]. Our study, for the first time, provided evidence regarding the modulatory effect of inflammation in prediabetes and the relative contribution of inflammation and prediabetes as well as their joint effect to the development of diabetes. Rather than a mere biomarker of prevalent or developing diabetes, elevated inflammation interacts with prediabetes and substantially contributes to an increased risk of incident diabetes. The stronger supra-additive interaction observed in participants younger than 60 years may reflect greater susceptibility to metabolic-inflammatory stress earlier in life, whereas in older adults the effect may be attenuated by competing comorbidities and mortality risks. Our previous findings of a synergistic effect between metabolic disorders and cumulative inflammation on diabetogenesis in young adults further support this hypothesis [35]. Nevertheless, these age-specific findings were exploratory and should be interpreted with caution. Collectively, our results lend epidemiological support to the hypothesis that inflammation may play a key role in driving the progression to diabetes in high-risk populations, highlighting the need for further mechanistic research.

Our findings of a super-additive interaction between elevated inflammation and prediabetes cannot establish causality due to the observational nature of the study, but they are suggestive of a biological correlation [36]. Elevated inflammation and prediabetes are likely to share some common mechanisms that synergistically lead to higher risks of incident diabetes. The onset of type 2 diabetes involves a multifaceted, complex pathogenesis, which is largely determined by the progressive failure of β-cells to compensate for the insulin demands in the context of existing insulin resistance (IR) [2,6]. IR exists for years before diabetes and throughout the development of diabetes. Diminished β-cell function is prominent in prediabetes and underlies the progression to diabetes [2,18]. In the prediabetic milieu, the failure and apoptosis of β-cells should result from a hyperglycemia-driven, IL-1β-induced inflammatory process in islet cells, mechanically involving high expression of the proapoptotic receptor on β-cells [37,38]. Additionally, pathological changes in oxidative stress, endoplasmic reticulum stress, and/or ectopic lipid deposition in the pancreas should deteriorate the insulin secretory function of β-cells by either activating the proinflammatory sensor of metabolism or being exaggerated by the inflammatory response, resulting in abnormal islet β-cell biology and even cell apoptosis [39]. Indeed, histologic changes characteristic of inflammation (*e.g*. immune cell infiltration [40], amyloid deposition [5], cell death, and fibrosis [41]) have been noted within the islets of subjects with type 2 diabetes. Moreover, there is likely biological interplay between CRP production and insulin action in hepatic cells. Impaired insulin sensitivity may lead to enhanced CRP expression by counteracting the physiological effect of insulin on hepatic acute-phase protein synthesis [42].

Although prediabetes represents an enhanced risk of incident diabetes, reversion to normoglycemia remains possible. From a perspective of public health, the additivity of effects is important for public health interventions and clinical decision making, providing valuable information for identifying high-risk populations and prioritise preventive measures [43], For prediabetic individuals, the cornerstone of diabetes prevention is lifestyle modification that includes weight loss and exercise [44], with intensive programmes achieving a 40–70% relative risk reductions for incident diabetes. It is noteworthy that lifestyle management per se results in decreased inflammatory levels. In addition, a large body of tantalising data supports ongoing investigations of multiple therapies to target inflammation to improve cardiometabolic health [45]. Emerging evidence indicates that some glucose-lowering agents, such as metformin [44], glucagon-like 1 analogs [46] and sodium-dependent glucose transporter 2 inhibitors [47], may reduce diabetes incidence in prediabetic individuals, possibly through combined effects on body weight and inflammation [44]. In addition, clinical studies have explored anti-inflammatory strategies, such as antagonism of tumor necrosis factor (TNF)-α (13) and IL-1β [14,16,17], which have been shown to improve β-cell function and glucose homeostasis, with or without improving IR [19,48]. A recent meta-analysis comprising 2921 individuals demonstrated a significant overall HbA1c-lowering effect of IL-1 antagonism [20]. While our observational results cannot justify clinical recommendations, they are consistent with and complementary to this broader body of evidence. It is therefore reasonable to postulate that anti-inflammatory pharmacotherapy may represent a promising adjunctive approach to lifestyle management in preventing diabetes among high-risk subgroups.

Importantly, hsCRP may have potential utility in risk stratification for individuals with prediabetes. In our analyses, the super-additive interaction between elevated hsCRP and prediabetes was consistent across thresholds of 1, 2, and 3 mg/L, underscoring the robustness of the findings. However, the optimal cut-off for clinical translation (*e.g*. targeted pharmacologic or lifestyle interventions) may differ across populations, highlighting the need for further research to define population-specific thresholds and evaluate their practical utility. Thus, further well-designed diabetes-focused studies are warranted to examine the potential of hsCRP as a biomarker and of anti-inflammatory therapies as preventive strategies in prediabetes.

The major strengths of our study include the prospective cohort design, large sample size, and long-term follow-up as well as the comprehensive investigation of the interaction between prediabetes and inflammation on both multiplicative and additive scales and across different age and sex subgroups. To our knowledge, this is the first study to investigate how elevated inflammation modifies the risks of incident type 2 diabetes in prediabetes and to quantify their relative contribution to developing diabetes, providing epidemiological evidence for the potential translation of anti-inflammatory, risk-reducing therapies to clinical practice.

Limitations of the current study should be noted. First, diabetes status was defined using a combination of self-reported physician diagnosis, use of antidiabetic medication, and a single FBG measurement, with IFG used as the operational definition of prediabetes, without confirmatory 2-hour plasma glucose during a 75-g oral glucose tolerance test (OGTT) or HbA1c assessment. Although this pragmatic approach is commonly applied in large-scale, community-based cohorts where repeated OGTT or HbA1c testing is rarely feasible in real-world settings [30,49–51], it may lead to misclassification of glycemic status and affect the accuracy of risk estimates. Nonetheless, the use of simple and widely available biomarkers makes our findings relevant to diverse populations, particularly in low-resource settings where more complex diagnostic tests are not routinely available. Second, we cannot distinguish type 2 from type 1 diabetes. However, the chance of misclassification is minimal because of the greater age of the study participants than the usual type 1 diabetes onset age and the predominance of type 2 diabetes (>95%) in all-cause diabetes. Third, although the analytic cohort was somewhat younger and healthier than those excluded, these were pre-specified, design-driven criteria that may limit generalisability but are unlikely to substantially compromise the internal validity of the observed associations. Fourth, as with all observational studies, residual confounding, including unmeasured factors such as diet, psychosocial stress, and over-the-counter inflammatory medication use, may persist and limit causal inference. Fifth, although hsCRP was measured at a single time point, consistent with previous studies [16,25,26], residual day-to-day variation could not be entirely excluded. Sixth, the participants were Han Chinese from a community-based cohort in northern China, which limits the generalisability of our findings to other populations, particularly other racial or ethnic groups. Additionally, the male skewness is prominent in our study, with approximately 80% of participants being men. This reflects the occupational profile of the study population, as the Kailuan cohort is based in a coal-mining city where the workforce is predominantly male manual laborers. Although sex-specific analyses yielded generally consistent associations between hsCRP, IFG, and diabetes risk in both men and women, sex differences in inflammatory responses and glucose metabolism reported in previous studies could influence the magnitude of these associations. Further validation in more gender-balanced populations and different geographic or occupational settings is warranted to enhance external generalisability.

## CONCLUSIONS

Collectively, in a population-based, prospective cohort in China, elevated inflammation in prediabetes supra-additively led to a higher risk of incident type 2 diabetes, and the effect upon clustering of both risk factors was higher than the addition of the risks associated with each individual factor. Our findings provide epidemiological evidence suggesting that inflammation may play a contributory role in diabetes progression. While these results cannot establish causality, they highlight high-risk subgroups and underscore the need for further mechanistic and interventional studies to evaluate the potential of anti-inflammatory approaches in diabetes prevention.

## Additional material


Online Supplementary Document
