# Supplementary material for: Elevated inflammation supra-additively promotes the progression from prediabetes to diabetes: a prospective cohort study
Source: J Glob Health. 2025 Oct 24;15:04318. doi: 10.7189/jogh.15.04318 (PMC12550539; doi:10.7189/jogh.15.04318)
Supplement: Online Supplementary Document [file jogh-15-04318-s001.pdf]

Supplement to: Lan Y, Wu D, Zheng H, Ding X, Zhou H, Wu K, Wu W, Huang Z, Wang X, Wang W, Wu S, Chen Y. Elevated inflammation supra-additively promotes the progression from prediabetes to diabetes: a prospective cohort study. J Glob Health. 2025;15:04318.

eTable 1. Baseline difference of the included and excluded individuals in the study cohort 3

eTable 2. Baseline characteristics of the study participants acoss IFG status (raw data) 5

eTable 3. Association between hsCRP levels and incident type 2 diabetes (Cut-off point: 2mg/L) 7

eTable 4. Association between IFG (WHO criteria) and incident type 2 diabetes (14215/82475) 8

eTable 5. IFG (WHO criteria)-associated risk of incident type 2 diabetes in the entire cohort and stratified by hsCRP strata (<2, >2mg/L) 9

eTable 6. Association between co-exposure to IFG (WHO criteria) and hsCRP≥2 mg/L and incident type 2 diabetes in the entire cohort 10

eTable 7. Association between hsCRP levels and incident type 2 diabetes (Cut-off point: 1mg/L and 3mg/L) 11

eTable 8. IFG (WHO criteria)-associated risk of incident type 2 diabetes in the entire cohort and stratified by hsCRP strata (<1, 1-3, >3mg/L) 12

eTable 9. Association between hsCRP levels and incident type 2 diabetes (Cut-off point: 3mg/L) 14

eTable 10. IFG (WHO criteria)-associated risk of incident type 2 diabetes in the entire cohort and stratified by hsCRP strata (<3, ≥3 mg/L) 15

eTable 11. Additive effect of IFG (WHO criteria) and hsCRP≥3 mg/L on 11-year type 2 diabetes risks 16

eTable 12. Association between co-exposure to IFG (WHO criteria) and hsCRP≥3 mg/L and incident type 2 diabetes in the entire cohort 17

eTable 13. Association between co-exposure to IFG (WHO criteria) and hsCRP≥1 mg/L and incident type 2 diabetes in the entire cohort 18

eTable 14. Additive effect of IFG (WHO criteria) and hsCRP≥1 mg/L on 11-year type 2 diabetes risks 19

eTable 15. Association between IFG (ADA criteria) and incident type 2 diabetes 20

eTable 16. IFG (ADA criteria)-associated risk of incident type 2 diabetes in the entire cohort and stratified by hsCRP strata (<2, >2mg/L) 21

eTable 17. Association between co-exposure to IFG (ADA criteria) and hsCRP≥2 mg/L and incident type 2 diabetes in the entire cohort 22

eTable 18. IFG (ADA criteria)-associated risk of incident type 2 diabetes in the entire cohort and stratified by hsCRP strata (<3, ≥3 mg/L) 23

eTable 19. Association between co-exposure to IFG (ADA criteria) and hsCRP≥3 mg/L and incident type 2 diabetes in the entire cohort 24

eTable 20. Additive effect of IFG (ADA criteria) and hsCRP≥3 mg/L on type 2 diabetes risks 25

eTable 21. Sensitivity analysis of association between co-exposure to IFG and hsCRP≥2 and incident type 2 diabetes by excluding individuals with preexisting CVD 26

eTable 22. Sensitivity analysis of association between co-exposure to IFG and hsCRP≥2 and incident type 2 diabetes by excluding individuals with suspected infection 27

eTable 23. Sensitivity analysis of association between co-exposure to IFG and hsCRP≥2 and incident type 2 diabetes on raw data (14175/82244) 28

eTable 24. Additive effect of IFG and hsCRP≥2 mg/L on 11-year type 2 diabetes risks in sensitivity analyses 29

eTable 25. Association between co-exposure to IFG and hsCRP≥2 mg/L and incident type 2 diabetes across sex subgroups 30

eTable 26. Association between co-exposure to IFG and hsCRP≥2 mg/L and incident type 2 diabetes across age subgroups (<60, ≥60 years) 31

eTable 27. Additive effect of IFG and hsCRP≥2 mg/L on type 2 diabetes risks across sex and age subgroups 32

eTable 28. Additive effect of IFG and hsCRP≥2 mg/L on type 2 diabetes risks across combined sex-age subgroups 33

eFig 1. Flowchart of the study participants 34

eFig 2. Dose–dependent association between log-transformed hsCRP and incident type 2 diabetes among the overall population (A), non-prediabetes (B), and prediabetes (C) 35

eTable 1. Baseline difference of the included and excluded individuals in the study cohort

	Include (n=8,2475)	Exclude (n=1,9035)	*P*-difference
Diabetes, No. (%)	0 (0.0)	8725 (45.8)	<0.0001
CVD, No. (%)	2467 (3.0)	1335 (7.0)	<0.0001
Age, mean (SD), years	50.4±12.0	58.0±13.2	<0.001
Male, No. (%)	66257 (80.3)	14608 (76.7)	<0.001
BMI, mean (SD), kg/m2	25.0±3.4	25.4±3.7<br>MissingN=674	<0.001
Waist circumference, mean (SD), cm	86.6±9.7<br>MissingN=121	89.1±10.0<br>MissingN=1005	<0.001
hsCRP, median (IQR), mg/L	0.8 (0.3–2.1)	1.2 (0.4–3.1)<br>MissingN=3186	<0.001
SBP, mean (SD), mm Hg,	129.7±20.4<br>MissingN=380	137.3±22.7<br>MissingN=812	<0.001
DBP, median (IQR), mm Hg,	80.0 (78.0–90.0)<br>MissingN=418	80.0 (78.0–90.0)<br>MissingN=831	<0.001
HDL-C, median (IQR), mmol/L,	1.5 (1.3–1.8)<br>MissingN=40	1.5 (1.3–1.8)<br>MissingN=1193	<0.001
LDL-C, mean (SD), mmol/L,	2.3±0.9<br>MissingN=135	2.4±0.9<br>MissingN=1435	<0.001
TC, mean (SD), mmol/L,	4.9±1.1<br>MissingN=17	5.0±1.2<br>MissingN=1180	<0.001
TG, median (IQR), mmol/L,	1.3 (0.9–1.9)<br>MissingN=77	1.3 (0.9–1.9)<br>MissingN=1210	<0.001
eGFR, median (IQR), ml/min/1.73m2,	82.1 (68.8–96.6)<br>MissingN=300	75.9 (62.7–90.5)<br>MissingN=1286	<0.001
Family history of diabetes, No. (%)	6842 (8.3)	1515 (8.0)	0.128
Education, No. (%)	MissingN=48	MissingN=992	<0.0001
Less than high school	65164 (79.1)	14831 (82.2)	
High school and above	17263 (20.9)	3212 (17.8)	
Drinking habits, No. (%),	MissingN=83	MissingN=812	<0.0001
Current drinker,	31352 (38.0)	5858 (32.1)	
Non-current drinker	51040 (62.0)	12365 (67.9)	
Smoking habits, No. (%),	MissingN=82	MissingN=813	<0.0001
Never smoker	49227 (59.8)	11020 (60.5)	
Ever smoker	4471 (5.4)	1510 (8.3)	
Current smoker	28695 (34.8)	5692 (31.2)	
Physical activities, No. (%),	MissingN=110	MissingN=1091	<0.0001
Low	7508 (9.1)	1537 (8.6)	
Moderate	62219 (75.5)	13173 (73.4)	
High	12638 (15.3)	3234 (17.9)	

Abbreviations: BMI: body mass index; hsCRP: high-sensitivity C-reactive protein; HDL-C: high-

density lipoprotein cholesterol; LDL-C: low-density lipoprotein cholesterol; TC: total cholesterol; TG: triglyceride; SBP: systolic blood pressure, DBP: diastolic blood pressure; eGFR: estimated glomerular filtration rate.

eTable 2. Baseline characteristics of the study participants acoss IFG status (raw data)

	**IFG (n= 7353)**	**Non-IFG (n=75,122)**
Age, mean (SD), years	52.4±10.7	50.2±12.1
Male, No. (%)	6391 (86.9)	59866 (79.7)
BMI, mean (SD), kg/m^2	25.9±3.4	24.9±3.4
Waist circumference, mean (SD), cm, MissingN=121	88.6±9.6	86.4±9.7
hsCRP, median (IQR), mg/L	0. 9 (0.4–2.4)	0.7 (0.3–2.0)
hsCRP-categories, No. (%)		
<2 mg/L	5209 (70.8)	55851 (74.4)
≥2mg/L	2144 (29.2)	19271 (25.6)
SBP, mean (SD), mm Hg, MissingN=380	135.7±21.0	129.1±20.3
DBP, median (IQR), mm Hg, MissingN=418	83.3 (80.0–91.0)	80.0 (77.0–90.0)
HDL-C, median (IQR), mmol/L, MissingN=40	1.5 (1.3–1.8)	1.5 (1.3–1.8)
LDL-C, mean (SD), mmol/L, MissingN=135	2.5±0.9	2.3±0.9
TC, mean (SD), mmol/L, MissingN=17	5.1±1.2	4.9±1.1
TG, median (IQR), mmol/L, MissingN=77	1.5 (1.0–2.2)	1.2 (0.9–1.8)
eGFR, median (IQR), ml/min/1.73m^2, MissingN=300	83.1 (69.0–98.9)	82.1 (68.9–96.4)
Creatine, median (IQR), umol/L, MissingN=300	88.0 (73.1–102.2)	89.0 (76.0–102.8)
Family history of diabetes, No. (%)	731 (9.9)	6111 (8.1)
Education, No. (%), MissingN=48		
Less than high school	6035 (82.1)	59129 (78.7)
High school and above	1313 (17.9)	15950 (21.2)
Drinking habits, No. (%), MissingN=83		
Current drinker,	3090 (42.1)	28262 (37.7)
Non-current drinker,	4256 (57.9)	46784 (62.3)
Smoking habits, No. (%), MissingN=82		
Never smoker	4094 (55.7)	45133 (60.1)
Ever smoker	482 (6.6)	3989 (5.3)
Current smoker	2770 (37.7)	25925 (34.6)
Physical activities, No. (%), MissingN=110		
Low	812 (11.1)	6696 (8.9)
Moderate	5283 (72.0)	56936 (75.8)
High	1243 (16.9)	11395 (15.2)
CVD, No. (%)	2220 (3.0)	247 (3.4)
Medication use, No. (%)		
Antihypertensives	294 (4.0)	1953 (2.6)
Statin	19 (0.3)	157 (0.2)
Fibrate	9 (0.12)	54 (0.07)

Abbreviations: BMI: body mass index; hsCRP: high-sensitivity C-reactive protein; HDL-C: high-

density lipoprotein cholesterol; LDL-C: low-density lipoprotein cholesterol; TC: total cholesterol; TG: triglyceride; SBP: systolic blood pressure, DBP: diastolic blood pressure; eGFR: estimated glomerular filtration rate.

eTable 3. Association between hsCRP levels and incident type 2 diabetes (Cut-off point: 2mg/L)

	**HRs (95% CIs)**		***P*-trend**	**Per SD**
	**hsCRP<2 mg/L**	**hsCRP≥2 mg/L**		
Entire population				
Event/Total	9801/61060	4414/21415		
Incidence rate	16.57	21.56		
Crude model	Reference	1.33 (1.29–1.38)	<0.0001	1.19 (1.17–1.21)
Model 1	Reference	1.27 (1.22–1.32)	<0.0001	1.16 (1.14–1.18)
Model 2	Reference	1.21 (1.16–1.25)	<0.0001	1.12 (1.10–1.14)
Model 3	Reference	1.12 (1.08–1.16)	<0.0001	1.06 (1.05–1.08)
Model 4	Reference	1.12 (1.08–1.16)	<0.0001	1.07 (1.06–1.09)

Model 1: adjusted for sex, age, smoking habits, alcohol consumption, physical activities, education, family history of diabetes, antihypertensives, and lipid-lowering drugs.

Model 2: further adjusted for *log (*TG/HDL-C) (continuous), blood pressure categories (non-hypertension, hypertension grade I, grade II, grade III), and eGFR (≥90, 60-90, 30-60, <30 ml/min/1.73m^2).

Model 3: Model 2+BMI (continuous).

Model 4: Model 2+ waist circumference (continuous).

eTable 4. Association between IFG (WHO criteria) and incident type 2 diabetes (14215/82475)

	**HRs (95% CIs)**		***P*-trend**	**Per SD for FBG**
	**Non-IFG**	**IFG**		
Entire population				
Event/Total	10565/75122	3650/7353		
Incidence rate	14.27	73.02		
Crude model	Reference	5.05 (4.86–5.24)	<0.0001	2.12 (2.09–2.16)
Model 1	Reference	4.87 (4.69–5.06)	<0.0001	2.10 (2.06–2.13)
Model 2	Reference	4.48 (4.31–4.66)	<0.0001	2.02 (1.99–2.05)
Model 3	Reference	4.34 (4.17–4.51)	<0.0001	1.99 (1.96–2.02)
Model 4	Reference	4.48 (4.31–4.65)	<0.0001	2.02 (1.98–2.05)

Model 1: adjusted for sex, age, smoking habits, alcohol consumption, physical activities, education, family history of diabetes, antihypertensives, and lipid-lowering drugs.

Model 2: further adjusted for *log (*TG/HDL-C) (continuous), blood pressure categories (non-hypertension, hypertension grade I, grade II, grade III), and eGFR (≥90, 60-90, 30-60, <30 ml/min/1.73m^2).

Model 3: Model 2+BMI (continuous).

Model 4: Model 2+ waist circumference (continuous).

eTable 5. IFG (WHO criteria)-associated risk of incident type 2 diabetes in the entire cohort and stratified by hsCRP strata (<2, >2mg/L)

	**HRs (95% CIs)**		***P*-trend**	***P*-INTm**
	**Non-IFG**	**IFG**		
**hsCRP<2 mg/L**				0.3451
Event/Total	7347/55851	2454/5209		
Incidence rate	13.25	66.76		
Crude model	Reference	5.00 (4.78–5.24)	<0.0001	
Model 1	Reference	4.78 (4.56–5.00)	<0.0001	
Model 2	Reference	4.40 (4.20–4.61)	<0.0001	
Model 3	Reference	4.25 (4.06–4.46)	<0.0001	
Model 4	Reference	4.41 (4.21–4.62)	<0.0001	
**hsCRP≥2 mg/L**				
Event/Total	3218/19271	1196/2144		
Incidence rate	17.35	90.45		
Crude model	Reference	5.08 (4.75–5.43)	<0.0001	
Model 1	Reference	5.01 (4.69–5.36)	<0.0001	
Model 2	Reference	4.67 (4.37–5.00)	<0.0001	
Model 3	Reference	4.52 (4.22–4.83)	<0.0001	
Model 4	Reference	4.62 (4.32–4.95)	<0.0001	

Model 1: adjusted for sex, age, smoking habits, alcohol consumption, physical activities, education, family history of diabetes, antihypertensives, and lipid-lowering drugs.

Model 2: further adjusted for *log (*TG/HDL-C) (continuous), blood pressure categories (non-hypertension, hypertension grade I, grade II, grade III), and eGFR (≥90, 60-90, 30-60, <30 ml/min/1.73m^2).

Model 3: Model 2+BMI (continuous).

Model 4: Model 2+ waist circumference (continuous).

P-INTm: IFG status (yes or not) * hsCRP subgroup (<2, ≥2)=0.3451; IFG status (yes or not) * *log*hsCRP=0.2962.

eTable 6. Association between co-exposure to IFG (WHO criteria) and hsCRP≥2 mg/L and incident type 2 diabetes in the entire cohort

	**Combination of IFG or not and hsCRP, HRs (95% CIs)**				***P*-trend**
	**hsCRP <2 mg/L & non-IFG**	**hsCRP ≥2 mg/L & non-IFG**	**hsCRP <2 mg/L & IFG**	**hsCRP ≥2 mg/L & IFG**	
Entire population					
Event/Total	7347/55851	3218/19271	2454/5209	1196/2144	
Incidence rate	13.25	17.35	66.76	90.45	
Crude model	Reference	1.31 (1.25–1.36)	4.98 (4.76–5.21)	6.69 (6.29–7.11)	<0.0001
Model 1	Reference	1.24 (1.19–1.30)	4.80 (4.59–5.03)	6.20 (5.83–6.59)	<0.0001
Model 2	Reference	1.20 (1.15–1.25)	4.44 (4.24–4.65)	5.53 (5.18–5.87)	<0.0001
Model 3	Reference	1.11 (1.07–1.16)	4.29 (4.09–4.49)	4.96 (4.66–5.28)	<0.0001
Model 4	Reference	1.11 (1.06–1.16)	4.45 (4.25–4.66)	5.06 (5.75–5.38)	<0.0001

P-INTm: IFG status (yes or not) * hsCRP subgroup (<2, ≥2 mg/L) = 0.3451; IFG status (yes or not) * loghsCRP=0.2962.

Model 1: adjusted for sex, age, smoking habits, alcohol consumption, physical activities, education, family history of diabetes, antihypertensives, and lipid-lowering drugs.

Model 2: further adjusted for *log (*TG/HDL-C) (continuous), blood pressure categories (non-hypertension, hypertension grade I, grade II, grade III), and eGFR (≥90, 60-90, 30-60, <30 ml/min/1.73m^2).

Model 3a: Model 2+BMI (continuous).

Model 4a: Model 2+ waist circumference (continuous).

eTable 7. Association between hsCRP levels and incident type 2 diabetes (Cut-off point: 1mg/L and 3mg/L)

	HRs (95% CIs)			***P*-trend**
	**hsCRP<1 mg/L**	**1≤hsCRP<3 mg/L**	**hsCRP≥3 mg/L**	
Entire population				
Event/Total	7064/46786	4021/20254	3130/15435	
Incidence rate	15.43	21.33	21.76	
Crude model	Reference	1.38 (1.33–1.43)	1.40 (1.35–1.46)	<0.0001
Model 1	Reference	1.33 (1.28–1.38)	1.32 (1.26–1.37)	<0.0001
Model 2	Reference	1.23 (1.18–1.28)	1.24 (1.19–1.29)	<0.0001
Model 3	Reference	1.12 (1.08–1.16)	1.13 (1.08–1.18)	<0.0001
Model 4	Reference	1.16 (1.11–1.20)	1.12 (1.08–1.17)	<0.0001

Model 1: adjusted for sex, age, smoking habits, alcohol consumption, physical activities, education, family history of diabetes, antihypertensives, and lipid-lowering drugs.

Model 2: further adjusted for *log (*TG/HDL-C) (continuous), blood pressure categories (non-hypertension, hypertension grade I, grade II, grade III), and eGFR (≥90, 60-90, 30-60, <30 ml/min/1.73m^2).

Model 3: Model 2+BMI (continuous).

Model 4: Model 2+ waist circumference (continuous).

eTable 8. IFG (WHO criteria)-associated risk of incident type 2 diabetes in the entire cohort and stratified by hsCRP strata (<1, 1-3, >3mg/L)

	**HRs (95% CIs)**		***P*-trend**	**Per SD in FBG**	***P*-INTm**
	**Non-IFG**	**IFG**			
**hsCRP<1 mg/L**					0.2994
Event/Total	5367/43025	1697/3761			
Incidence rate	12.47	62.05			
Crude model	Reference	4.94 (4.68–5.22)	<0.0001	2.14 (2.08–2.19)	
Model 1	Reference	4.72 (4.67–4.99)	<0.0001	2.10 (2.05–2.15)	
Model 2	Reference	4.33 (4.10–4.58)	<0.0001	2.02 (1.98–2.07)	
Model 3	Reference	4.17 (3.95–4.41)	<0.0001	1.99 (1.94–2.04)	
Model 4	Reference	4.34 (4.11–4.59)	<0.0001	2.02 (1.97–2.07)	
**1≤hsCRP<3 mg/L**					
Event/Total	2908/18173	1113/2081			
Incidence rate	16.60	83.57			
Crude model	Reference	4.98 (4.64–5.33)	<0.0001	2.13 (2.06–2.19)	
Model 1	Reference	4.85 (4.52–5.20)	<0.0001	2.11 (2.04–2.18)	
Model 2	Reference	4.59 (4.26–4.91)	<0.0001	2.05 (1.99–2.12)	
Model 3	Reference	4.48 (4.18–4.81)	<0.0001	2.03 (1.97–2.10)	
Model 4	Reference	4.58 (4.27–4.92)	<0.0001	2.05 (1.99–2.12)	
**hsCRP≥3 mg/L**					
Event/Total	2290/13924	840/1511			
Incidence rate	17.02	90.17			
Crude model	Reference	5.15 (4.75–5.57)	<0.0001	2.05 (1.98–2.12)	
Model 1	Reference	5.10 (4.72–5.53)	<0.0001	2.04 (1.98–2.11)	

Model 2	Reference	4.71 (4.35–5.11)	<0.0001	1.98 (1.92–2.05)	
Model 3	Reference	4.55 (4.20–4.94)	<0.0001	1.95 (1.89–2.02)	
Model 4	Reference	4.66 (4.30–5.05)	<0.0001	1.97 (1.91–2.04)	

Model 1: adjusted for sex, age, smoking habits, alcohol consumption, physical activities, education, family history of diabetes, antihypertensives, and lipid-lowering drugs.

Model 2: further adjusted for *log (*TG/HDL-C) (continuous), blood pressure categories (non-hypertension, hypertension grade I, grade II, grade III), and eGFR (≥90, 60-90, 30-60, <30 ml/min/1.73m^2).

Model 3: Model 2+BMI (continuous).

Model 4: Model 2+ waist circumference (continuous).

eTable 9. Association between hsCRP levels and incident type 2 diabetes (Cut-off point: 3mg/L)

	**HRs (95% CIs)**		***P*-trend**
	**hsCRP<3 mg/L**	**hsCRP≥3 mg/L**	
Event/Total	11085/67040	3130/15435	
Incidence rate	17.15	21.76	
Crude model	Reference	1.26 (1.21–1.32)	<0.0001
Model 1	Reference	1.19 (1.15–1.24)	<0.0001
Model 2	Reference	1.15 (1.11–1.20)	<0.0001
Model 3	Reference	1.08 (1.04–1.12)	<0.0001
Model 4	Reference	1.06 (1.02–1.11)	<0.0001

Model 1: adjusted for sex, age, smoking habits, alcohol consumption, physical activities, education, family history of diabetes, antihypertensives, and lipid-lowering drugs.

Model 2: further adjusted for *log (*TG/HDL-C) (continuous), blood pressure categories (non-hypertension, hypertension grade I, grade II, grade III), and eGFR (≥90, 60-90, 30-60, <30 ml/min/1.73m^2).

Model 3: Model 2+BMI (continuous).

Model 4: Model 2+ waist circumference (continuous).

eTable 10. IFG (WHO criteria)-associated risk of incident type 2 diabetes in the entire cohort and stratified by hsCRP strata (<3, ≥3 mg/L)

	**HRs (95% CIs)**		***P*-trend**	***P*-INTm**
	**Non-IFG**	**IFG**		
**hsCRP<3 mg/L**				0.4492
Event/Total	8275/61198	2810/5842		
Incidence rate	13.66	69.10		
Crude model	Reference	5.01 (4.80–5.23)	<0.0001	
Model 1	Reference	4.81 (4.60–5.02)	<0.0001	
Model 2	Reference	4.43 (4.24–4.63)	<0.0001	
Model 3	Reference	4.23 (4.10–4.48)	<0.0001	
Model 4	Reference	4.44 (4.25–4.64)	<0.0001	
**hsCRP≥3 mg/L**				
Event/Total	2290/13924	840/1511		
Incidence rate	17.02	90.17		
Crude model	Reference	5.15 (4.75–5.57)	<0.0001	
Model 1	Reference	5.10 (4.71–5.53)	<0.0001	
Model 2	Reference	4.71 (4.35–5.11)	<0.0001	
Model 3	Reference	4.55 (4.20–4.94)	<0.0001	
Model 4	Reference	4.66 (4.30–5.05)	<0.0001	

Model 1: adjusted for sex, age, smoking habits, alcohol consumption, physical activities, education, family history of diabetes, antihypertensives, and lipid-lowering drugs.

Model 2: further adjusted for *log (*TG/HDL-C) (continuous), blood pressure categories (non-hypertension, hypertension grade I, grade II, grade III), and eGFR (≥90, 60-90, 30-60, <30 ml/min/1.73m^2).

Model 3: Model 2+BMI (continuous).

Model 4: Model 2+ waist circumference (continuous).

eTable 11. Additive effect of IFG (WHO criteria) and hsCRP≥3 mg/L on 11-year type 2 diabetes risks

**Main effects – hazard ratios**	**Model 1**	**Model 2**	**Model 3**	**Model 4**
IFG	4.82 (4.62–5.04)	4.46 (4.27–4.66)	4.31 (4.12–4.50)	4.46 (4.27–4.66)
hsCRP≥3 mg/L	1.18 (1.12–1.23)	1.15 (1.10–1.20)	1.08 (1.03–1.13)	1.09 (1.04–1.14)
Joint effect	5.93 (5.52–6.37)	5.29 (4.93–5.69)	4.81 (4.47–5.17)	4.97 (4.62–5.34)
RERI	0.93 (0.48–1.37)	0.68 (0.29–1.08)	0.42 (0.06–0.79)	0.42 (0.04–0.80)
AP	0.16 (0.09–0.22)	0.13 (0.06–0.20)	0.09 (0.02–0.16)	0.08 (0.01–0.16)
S	1.23 (1.12–1.35)	1.19 (1.08–1.31)	1.13 (1.02–1.24)	1.12 (1.01–1.23)
**Attributable proportion, %**				
IFG	77.48	80.65	86.88	87.15
hsCRP≥3 mg/L	3.65	3.50	2.10	2.27
Joint effect	18.87	15.85	11.02	10.58

Model 1: adjusted for sex, age, smoking habits, alcohol consumption, physical activities, education, family history of diabetes, antihypertensives, and lipid-lowering drugs.

Model 2: further adjusted for *log (*TG/HDL-C) (continuous), blood pressure categories (non-hypertension, hypertension grade I, grade II, grade III), and eGFR (≥90, 60-90, 30-60, <30 ml/min/1.73m^2).

Model 3: Model 2+BMI (continuous).

Model 4: Model 2+ waist circumference (continuous).

Abbreviations: RERI, relative excess risk due to interaction; AP, attributable proportion due to interaction; S, the synergy index; others are as eTable 1.

eTable 12. Association between co-exposure to IFG (WHO criteria) and hsCRP≥3 mg/L and incident type 2 diabetes in the entire cohort

	**Combination of IFG or not and hsCRP, HRs (95% CIs)**				***P*-trend**
	**hsCRP < 3mg/L & non-IFG**	**hsCRP ≥ 3mg/L & non-IFG**	**hsCRP < 3mg/L & IFG**	**hsCRP ≥ 3mg/L & IFG**	
Entire population					
Event/Total	8275/61198	2290/13924	2810/5842	840/1511	
Incidence rate	13.66	17.02	69.10	90.17	
Crude model	Reference	1.24 (1.19–1.30)	4.99 (4.78–5.21)	6.47 (6.03–6.95)	<0.0001
Model 1	Reference	1.78 (1.12–1.23)	4.82 (4.62–5.04)	5.93 (5.52–6.37)	<0.0001
Model 2	Reference	1.15 (1.10–1.20)	4.46 (4.27–4.66)	5.29 (4.93–5.69)	<0.0001
Model 3	Reference	1.08 (1.03–1.13)	4.31 (4.12–4.50)	4.81 (4.47–5.17)	<0.0001
Model 4	Reference	1.06 (1.01–1.11)	4.47 (4.28–4.67)	4.84 (4.50–5.20)	<0.0001

P-INTm: IFG status (yes or not) * hsCRP subgroup (<3, ≥3 mg/L) = 0.4492.

Model 1: adjusted for sex, age, smoking habits, alcohol consumption, physical activities, education, family history of diabetes, antihypertensives, and lipid-lowering drugs.

Model 2: further adjusted for *log (*TG/HDL-C) (continuous), blood pressure categories (non-hypertension, hypertension grade I, grade II, grade III), and eGFR (≥90, 60-90, 30-60, <30 ml/min/1.73m^2).

Model 3a: Model 2+BMI (continuous).

Model 4a: Model 2+ waist circumference (continuous).

eTable 13. Association between co-exposure to IFG (WHO criteria) and hsCRP≥1 mg/L and incident type 2 diabetes in the entire cohort

	**Combination of IFG or not and hsCRP, HRs (95% CIs)**				***P*-trend**
	**hsCRP < 1 mg/L & non-IFG**	**hsCRP ≥ 1 mg/L & non-IFG**	**hsCRP < 1 mg/L & IFG**	**hsCRP ≥ 1 mg/L & IFG**	
Entire population					
Event/Total	53677/43025	5198/32097	1697/3761	1953/3592	
Incidence rate	12.45	16.76	61.95	86.11	
Crude model	Reference	1.34 (1.29–1.39)	4.92 (4.66–5.20)	6.79 (6.44–7.15)	<0.0001
Model 1	Reference	1.28 (1.24–1.34)	4.74 (4.49–5.01)	6.29 (5.97–6.64)	<0.0001
Model 2	Reference	1.21 (1.16–1.26)	4.36 (4.13–4.61)	5.54 (5.25–5.84)	<0.0001
Model 3	Reference	1.10 (1.06–1.15)	4.20 (3.97–4.44)	4.92 (4.67–5.19)	<0.0001
Model 4	Reference	1.09 (1.05–1.13)	4.23 (4.01–4.48)	4.87 (4.62–5.14)	<0.0001

P-INTm: IFG status (yes or not) * hsCRP subgroup (<1, ≥1 mg/L) =0.1298.

Model 1: adjusted for sex, age, smoking habits, alcohol consumption, physical activities, education, family history of diabetes, antihypertensives, and lipid-lowering drugs.

Model 2: further adjusted for *log (*TG/HDL-C) (continuous), blood pressure categories (non-hypertension, hypertension grade I, grade II, grade III), and eGFR (≥90, 60-90, 30-60, <30 ml/min/1.73m^2).

Model 3: Model 2+BMI (continuous).

Model 4: Model 2+ waist circumference (continuous).

eTable 14. Additive effect of IFG (WHO criteria) and hsCRP≥1 mg/L on 11-year type 2 diabetes risks

**Main effects – hazard ratios**	**Model 1**	**Model 2**	**Model 3**	**Model 4**
IFG	4.74 (4.49–5.01)	4.36 (4.13–4.61)	4.20 (3.97–4.44)	4.23 (4.01–4.48)
hsCRP≥1 mg/L	1.28 (1.24–1.34)	1.22 (1.16–1.26)	1.10 (1.06–1.15)	1.09 (1.05–1.13)
Joint effect	6.29 (5.97–6.64)	5.54 (5.25–5.84)	4.92 (4.67–5.19)	4.87 (4.62–5.14)
RERI	1.27 (0.91–1.63)	0.96 (0.64–1.29)	0.62 (0.32–0.92)	0.55 (0.25–0.85)
AP	0.20 (0.15–0.25)	0.17 (0.12–0.23)	0.13 (0.07–0.18)	0.11 (0.05–0.17)
S	1.32 (1.22–1.42)	1.26 (1.17–1.38)	1.19 (1.09–1.29)	1.16 (1.07–1.27)
**Attributable proportion, %**				
IFG	70.69	74.01	81.63	83.47
hsCRP≥1 mg/L	5.30	4.85	2.56	2.32
Joint effect	24.01	21.14	15.81	14.21

Model 1: adjusted for sex, age, smoking habits, alcohol consumption, physical activities, education, family history of diabetes, antihypertensives, and lipid-lowering drugs.

Model 2: further adjusted for *log (*TG/HDL-C) (continuous), blood pressure categories (non-hypertension, hypertension grade I, grade II, grade III), and eGFR (≥90, 60-90, 30-60, <30 ml/min/1.73m^2).

Model 3: Model 2+BMI (continuous).

Model 4: Model 2+ waist circumference (continuous).

Abbreviations: RERI, relative excess risk due to interaction; AP, attributable proportion due to interaction; S, the synergy index; others are as eTable 1.

eTable 15. Association between IFG (ADA criteria) and incident type 2 diabetes

	**HR (95% CIs)**		***P*-trend**
	**Non-IFG**	**IFG**	
Entire population			
Event/Total	7399/63587	6816/18888	
Incidence rate	11.57	45.30	
Crude model	Reference	3.88 (3.76–4.01)	<0.0001
Model 1	Reference	3.81 (3.69–3.94)	<0.0001
Model 2	Reference	3.57 (3.45–3.69)	<0.0001
Model 3	Reference	3.46 (3.35–3.58)	<0.0001
Model 4	Reference	3.56 (3.44–3.68)	<0.0001

IFG was redefined as fasting blood glucose between 5.6 and 6.9 mmol/L among non-diabetic individuals.

Model 1: adjusted for sex, age, smoking habits, alcohol consumption, physical activities, education, family history of diabetes, antihypertensives, and lipid-lowering drugs.

Model 2: further adjusted for *log (*TG/HDL-C) (continuous), blood pressure categories (non-hypertension, hypertension grade I, grade II, grade III) (non-hypertension, hypertension grade I, grade II, grade III), and eGFR (≥90, 60-90, 30-60, <30 ml/min/1.73m^2).

Model 3a: Model 2+BMI (continuous).

Model 4a: Model 2+ waist circumference (continuous).

eTable 16. IFG (ADA criteria)-associated risk of incident type 2 diabetes in the entire cohort and stratified by hsCRP strata (<2, >2mg/L)

	**HR (95% CIs)**		***P*-trend**	***P*-INTm**
	**Non-IFG**	**IFG**		
hsCRP<2 mg/L (9801/61060)				0.2920
Event/Total	5128/47250	4673/13810		
Incidence rate	10.71	41.48		
Crude model	Reference	3.85 (3.70–4.01)	<0.0001	
Model 1	Reference	3.75 (3.60–3.90)	<0.0001	
Model 2	Reference	3.52 (4.38–3.67)	<0.0001	
Model 3	Reference	3.41 (3.27–3.55)	<0.0001	
Model 4	Reference	3.51 (3.37–3.65)	<0.0001	
hsCRP≥2 mg/L (4414/21415)				
Event/Total	2271/16337	2143/5078		
Incidence rate	14.11	56.67		
Crude model	Reference	3.95 (3.72–4.19)	<0.0001	
Model 1	Reference	3.93 (3.71–4.17)	<0.0001	
Model 2	Reference	3.70 (3.48–3.93)	<0.0001	
Model 3	Reference	3.59 (3.38–3.81)	<0.0001	
Model 4	Reference	3.69 (4.47–3.91)	<0.0001	

IFG was redefined as fasting blood glucose between 5.6 and 6.9 mmol/L among non-diabetic individuals.

Model 1: adjusted for sex, age, smoking habits, alcohol consumption, physical activities, education, family history of diabetes, antihypertensives, and lipid-lowering drugs.

Model 2: further adjusted for *log (*TG/HDL-C) (continuous), blood pressure categories (non-hypertension, hypertension grade I, grade II, grade III), and eGFR (≥90, 60-90, 30-60, <30 ml/min/1.73m^2).

Model 3: Model 2+BMI (continuous).

Model 4: Model 2+ waist circumference (continuous).

eTable 17. Association between co-exposure to IFG (ADA criteria) and hsCRP≥2 mg/L and incident type 2 diabetes in the entire cohort

	**Combination of IFG or not and hsCRP, HRs (95% CIs)**				***P*-trend**
	**hsCRP <2 mg/L & non-IFG**	**hsCRP ≥2 mg/L & non-IFG**	**hsCRP <2 mg/L & IFG**	**hsCRP ≥2 mg/L & IFG**	
Entire population					
Event/Total	5128/47250	2271/16337	4673/13810	2143/5078	
Incidence rate	0.71	14.11	41.48	56.67	
Crude model	Reference	1.31 (1.25–1.38)	3.84 (3.69–4.00)	5.22 (4.96–5.49)	<0.0001
Model 1	Reference	1.24 (1.18–1.31)	3.76 (3.61–3.92)	4.88 (4.63–5.13)	<0.0001
Model 2	Reference	1.20 (1.14–1.26)	3.54 (3.40–3.69)	4.40 (4.18–4.64)	<0.0001
Model 3	Reference	1.12 (1.06–1.17)	3.42 (3.29–3.56)	3.97 (3.77–4.18)	<0.0001
Model 4	Reference	1.11 (1.06–1.17)	3.53 (3.39–3.67)	4.06 (3.86–4.28)	<0.0001

IFG was redefined as fasting blood glucose between 5.6 and 6.9 mmol/L among non-diabetic individuals.

P-INTm: IFG status (yes or not) * hsCRP subgroup (<2, ≥2) =0.2960 (Model 3); IFG status (yes or not) * *log*hsCRP=0.4785 (Model 3)

Model 1: adjusted for sex, age, smoking habits, alcohol consumption, physical activities, education, family history of diabetes, antihypertensives, and lipid-lowering drugs.

Model 2: further adjusted for *log (*TG/HDL-C) (continuous), blood pressure categories (non-hypertension, hypertension grade I, grade II, grade III), and eGFR (≥90, 60-90, 30-60, <30 ml/min/1.73m^2).

Model 3: Model 2+BMI (continuous).

Model 4: Model 2+ waist circumference (continuous

eTable 18. IFG (ADA criteria)-associated risk of incident type 2 diabetes in the entire cohort and stratified by hsCRP strata (<3, ≥3 mg/L)

	**HR (95% CIs)**		***P*-trend**	**Per SD in FBG**	***P*-INTm**
	**Non-IFG**	**IFG**			
**HsCRP<3 mg/L**					0.6071
Event/Total	5757/51709	5328/15331			
Incidence rate	11.02	42.99			
Crude model	Reference	3.88 (3.74–4.03)	<0.0001	2.14 (2.10–2.18)	
Model 1	Reference	3.78 (3.64–3.93)	<0.0001	2.11 (2.07–2.15)	
Model 2	Reference	3.56 (3.43–3.70)	<0.0001	2.04 (2.00–2.08)	
Model 3	Reference	3.44 (3.31–3.57)	<0.0001	2.01 (1.97–2.04)	
Model 4	Reference	3.54 (3.41–3.68)	<0.0001	2.04 (2.00–2.08)	
**HsCRP≥3 mg/L**					
Event/Total	1642/11878	1488/3557			
Incidence rate	14.00	56.11			
Crude model	Reference	3.93 (3.66–4.22)	<0.0001	2.05 (1.98–2.12)	
Model 1	Reference	3.91 (3.65–4.20)	<0.0001	2.04 (1.98–2.11)	
Model 2	Reference	3.66 (3.41–3.93)	<0.0001	1.98 (1.92–2.05)	
Model 3	Reference	3.55 (3.31–3.81)	<0.0001	1.95 (1.89–2.02)	
Model 4	Reference	3.66 (3.40–3.92)	<0.0001	1.97 (1.91–2.04)	

IFG was redefined as fasting blood glucose between 5.6 and 6.9 mmol/L among non-diabetic individuals.

Model 1: adjusted for sex, age, smoking habits, alcohol consumption, physical activities, education, family history of diabetes, antihypertensives, and lipid-lowering drugs.

Model 2: further adjusted for *log (*TG/HDL-C) (continuous), blood pressure categories (non-hypertension, hypertension grade I, grade II, grade III), and eGFR (≥90, 60-90, 30-60, <30 ml/min/1.73m^2).

Model 3: Model 2+BMI (continuous).

Model 4: Model 2+ waist circumference (continuous).

eTable 19. Association between co-exposure to IFG (ADA criteria) and hsCRP≥3 mg/L and incident type 2 diabetes in the entire cohort

	**Combination of IFG or not and hsCRP, HRs (95% CIs)**				***P*-trend**
	**hsCRP < 3mg/L & non-IFG**	**hsCRP ≥ 3mg/L & non-IFG**	**hsCRP < 3mg/L & IFG**	**hsCRP ≥ 3mg/L & IFG**	
Entire population					
Event/Total	5757/51709	1642/11878	5328/15331	1488/3557	
Incidence rate	11.02	14.00	42.99	56.11	
Crude model	Reference	1.27 (1.20–1.34)	3.87 (3.73–4.01)	5.02 (4.74–5.32)	<0.0001
Model 1	Reference	1.19 (1.13–1.26)	3.79 (3.65–3.94)	4.65 (4.39–4.93)	<0.0001
Model 2	Reference	1.17 (1.10–1.23)	3.57 (3.44–3.71)	4.24 (4.00–4.49)	<0.0001
Model 3	Reference	1.10 (1.04–1.16)	3.45 (3.32–3.58)	3.86 (3.64–4.09)	<0.0001
Model 4	Reference	1.08 (1.02–1.14)	3.55 (3.42–3.69)	3.91 (3.69–4.14)	<0.0001

P-INTm: IFG status (yes or not) * hsCRP subgroup (<3, ≥3) = 0.6071 (Model 3).

IFG was redefined as fasting blood glucose between 5.6 and 6.9 mmol/L among non-diabetic individuals.

Model 1: adjusted for sex, age, smoking habits, alcohol consumption, physical activities, education, family history of diabetes, antihypertensives, and lipid-lowering drugs.

Model 2: further adjusted for *log (*TG/HDL-C) (continuous), blood pressure categories (non-hypertension, hypertension grade I, grade II, grade III), and eGFR (≥90, 60-90, 30-60, <30 ml/min/1.73m^2).

Model 3: Model 2+BMI (continuous).

Model a: Model 2+ waist circumference (continuous).

eTable 20. Additive effect of IFG (ADA criteria) and hsCRP≥3 mg/L on type 2 diabetes risks

**Main effects – hazard ratios**	**Model 1**	**Model 2**	**Model 3**	**Model 4**
IFG	3.79 (3.65–3.94)	3.57 (3.44–3.71)	3.45 (3.32–3.58)	3.55 (3.42–3.69)
hsCRP≥3 mg/L	1.19 (1.13–1.26)	1.17 (1.10–1.23)	1.10 (1.04–1.16)	1.08 (1.02–1.14)
Joint effect	4.65 (4.39–4.93)	4.24 (4.00–4.49)	3.87 (4.64–4.09)	3.91 (3.69–4.14)
RERI	0.67 (0.41–0.94)	0.50 (0.26–0.74)	0.32 (0.09–0.54)	0.28 (0.05–0.51)
AP	0.14 (0.09–0.20)	0.12 (0.07–0.17)	0.08 (0.03–0.14)	0.07 (0.02–0.13)
S	1.23 (1.14–1.32)	1.18 (1.09–1.29)	1.12 (1.04–1.22)	1.11 (1.02–1.20)
**Attributable proportion, %**				
IFG	76.44	79.32	85.37	87.63
hsCRP≥3 mg/L	5.21	5.25	3.48	2.75
Joint effect	18.35	15.43	11.15	9.62

IFG was redefined as fasting blood glucose between 5.6 and 6.9 mmol/L among non-diabetic individuals.

Model 1: adjusted for sex, age, smoking habits, alcohol consumption, physical activities, education, family history of diabetes, antihypertensives, and lipid-lowering drugs.

Model 2: further adjusted for *log (*TG/HDL-C) (continuous), blood pressure categories (non-hypertension, hypertension grade I, grade II, grade III), and eGFR (≥90, 60-90, 30-60, <30 ml/min/1.73m^2).

Model 3: Model 2+BMI (continuous).

Model 4: Model 2+ waist circumference (continuous).

Abbreviations: RERI, relative excess risk due to interaction; AP, attributable proportion due to interaction; S, the synergy index; others are as eTable 1.

eTable 21. Sensitivity analysis of association between co-exposure to IFG and hsCRP≥2 and incident type 2 diabetes by excluding individuals with preexisting CVD

	Combination of IFG or not and hsCRP, HRs (95% CIs)				***P*-trend**
	**hsCRP <2 mg/L & non-IFG**	**hsCRP ≥2 mg/L & non-IFG**	**hsCRP <2 mg/L & IFG**	**hsCRP ≥2 mg/L & IFG**	
Entire population					
Event/Total	7068/54411	3059/18491	2360/5055	1136/2051	
Incidence rate	13.03	17.09	65.69	89.26	
Crude model	Reference	1.31 (1.25–1.36)	4.99 (4.76–5.23)	6.72 (6.31–7.16)	<0.0001
Model 1	Reference	1.25 (1.20–1.31)	4.80 (4.58–5.03)	6.26 (5.88–6.67)	<0.0001
Model 2	Reference	1.20 (1.15–1.26)	4.43 (4.23–4.65)	5.59 (5.25–5.96)	<0.0001
Model 3	Reference	1.12 (1.07–1.17)	4.28 (4.08–4.48)	5.03 (4.72–5.37)	<0.0001
Model 4	Reference	1.12 (1.07–1.17)	4.44 (4.24–4.66)	5.13 (4.81–5.47)	<0.0001

P-INTm: IFG status (yes or not) *hsCRP subgroup (<2, ≥2) = 0.2384; IFG status (yes or not) * *log*hsCRP=0.2461.

Model 1: adjusted for sex, age, smoking habits, alcohol consumption, physical activities, education, family history of diabetes, antihypertensives, and lipid-lowering drugs.

Model 2: further adjusted for *log (*TG/HDL-C) (continuous), blood pressure categories (non-hypertension, hypertension grade I, grade II, grade III), eGFR (≥90, 60-90, 30-60,<30 ml/min/1.73m^2).

Model 3: Model 2+BMI (continuous).

Model 4: Model 2+ waist circumference (continuous).

eTable 22. Sensitivity analysis of association between co-exposure to IFG and hsCRP≥2 and incident type 2 diabetes by excluding individuals with suspected infection

	**Combination of IFG or not and hsCRP, HRs (95% CIs)**				***P*-trend**
	**hsCRP <2 mg/L & non-IFG**	**hsCRP ≥2 mg/L & non-IFG**	**hsCRP <2 mg/L & IFG**	**hsCRP ≥2 mg/L & IFG**	
**Entire population**					
Event/Total	7347/55851	2748/16391	2454/5209	1035/1833	
Incidence rate	13.25	17.39	66.76	92.84	
Crude model	Reference	1.31 (1.25–1.37)	4.98 (4.76–5.22)	6.86 (6.43–7.32)	<0.0001
Model 1	Reference	1.25 (1.19–1.30)	4.80 (4.58–5.03)	6.36 (5.96–6.79)	<0.0001
Model 2	Reference	1.19 (1.14–1.25)	4.43 (4.22–4.64)	5.67 (5.31–6.06)	<0.0001
Model 3	Reference	1.11 (1.06–1.16)	4.28 (4.09–4.48)	5.08 (4.76–5.43)	<0.0001
Model 4	Reference	1.11 (1.06–1.16)	4.44 (4.24–4.65)	5.20 (4.86–5.55)	<0.0001

P-INTm: IFG status (yes or not) *hsCRP subgroup (<2, ≥2) = 0.1012; IFG status (yes or not) * *log*hsCRP=0.0302.

Model 1: adjusted for sex, age, smoking habits, alcohol consumption, physical activities, education, family history of diabetes, antihypertensives, and lipid-lowering drugs.

Model 2: further adjusted for *log (*TG/HDL-C) (continuous), blood pressure categories (non-hypertension, hypertension grade I, grade II, grade III), and eGFR (≥90, 60-90, 30-60, <30 ml/min/1.73m^2).

Model 3a: Model 2+BMI (continuous).

Model 4a: Model 2+ waist circumference(continuous).

eTable 23. Sensitivity analysis of association between co-exposure to IFG and hsCRP≥2 and incident type 2 diabetes on raw data (14175/82244)

	**Combination of IFG or not and hsCRP, HRs (95% CIs)**				***P*-trend**
	**hsCRP <2 mg/L & non-IFG**	**hsCRP ≥2 mg/L & non-IFG**	**hsCRP <2 mg/L & IFG**	**hsCRP ≥2 mg/L & IFG**	
**Entire population**					
Event/Total	7347/55851	3218/19271	2454/5209	1196/2144	
Incidence rate	13.25	17.35	66.76	90.45	
Crude model	Reference	1.31 (1.25–1.36)	4.98 (4.76–5.21)	6.69 (6.29–7.11)	<0.0001
Model 1	Reference	1.25 (1.19–1.30)	4.80 (4.59–5.03)	6.22 (5.85–6.62)	<0.0001
Model 2	Reference	1.20 (1.15–1.25)	4.44 (4.24–4.65)	5.53 (5.19–5.88)	<0.0001
Model 3	Reference	1.11 (1.07–1.16)	4.29 (4.09–4.49)	4.96 (4.66–5.28)	<0.0001
Model 4	Reference	1.11 (1.06–1.16)	4.46 (4.25–4.67)	5.06 (4.75–5.39)	<0.0001

P-INTm: IFG status (yes or not) *hsCRP subgroup (<2, ≥2) = 0.3615; IFG status (yes or not) * *log*hsCRP = 0.5490.

Model 1: adjusted for sex, age, smoking habits, alcohol consumption, physical activities, education, family history of diabetes, antihypertensives, and lipid-lowering drugs.

Model 2: further adjusted for *log (*TG/HDL-C) (continuous), blood pressure categories (non-hypertension, hypertension grade I, grade II, grade III), and eGFR (≥90, 60-90, 30-60, <30 ml/min/1.73m^2).

Model 3a: Model 2+BMI (continuous).

Model 4a: Model 2+ waist circumference(continuous).

eTable 24. Additive effect of IFG and hsCRP≥2 mg/L on 11-year type 2 diabetes risks in sensitivity analyses

**Main effects – hazard ratios**	**Excluding hsCRP≥10 mg/L**	**Excluding baseline CVD**	**Raw data**
IFG	4.28 (4.09–4.48)	4.28 (4.08–4.48)	4.29 (4.09–4.49)
hsCRP≥2 mg/L	1.11 (1.06–1.16)	1.12 (1.07–1.17)	1.11 (1.07–1.16)
Joint effect	5.09 (4.76–5.43)	5.04 (4.72–5.37)	4.96 (4.66–5.28)
RERI	0.70 (0.34–1.06)	0.64 (0.29–0.98)	0.56 (0.23–0.89)
AP	0.14 (0.07–0.20)	0.13 (0.06–0.19)	0.11 (0.05–0.18)
S	1.21 (1.10–1.32)	1.19 (1.09–1.30)	1.17 (1.07–1.27)
**Attributable proportion, %**			
IFG	80.20	81.19	83.08
hsCRP≥2 mg/L	2.69	2.97	2.78
Joint effect	17.11	15.84	14.14

All models were adjusted for sex, age, smoking habits, alcohol consumption, physical activities, education, family history of diabetes, antihypertensives, lipid-lowering drugs, *log (*TG/HDL-C) (continuous), blood pressure categories (non-hypertension, hypertension grade I, grade II, grade III), eGFR (≥90, 60-90, 30-60, <30 ml/min/1.73m^2), and BMI (continuous).

Abbreviations: RERI, relative excess risk due to interaction; AP, attributable proportion due to interaction; S, the synergy index; others are as eTable 1

eTable 25. Association between co-exposure to IFG and hsCRP≥2 mg/L and incident type 2 diabetes across sex subgroups

	**Combination of IFG or not and hsCRP, HRs (95% CIs)**				***P*-trend**	***P-INTm***
	**hsCRP <2 mg/L & non-IFG**	**hsCRP ≥2 mg/L & non-IFG**	**hsCRP <2 mg/L & IFG**	**hsCRP ≥2 mg/L & IFG**		
**Male**						<0.0001
Event/Total	6108/44779	2489/15087	2141/4617	938/1774		
Incidence rate	13.96	17.42	65.73	83.85		
Crude model	Reference	1.24 (1.19–1.30)	4.65 (4.43–4.89)	5.88 (5.49–6.30)	<0.0001	
Model 1	Reference	1.20 (1.15–1.26)	4.56 (4.34–4.79)	5.61 (5.23–6.01)	<0.0001	
Model 2	Reference	1.17 (1.11–1.22)	4.26 (4.06–4.48)	5.11 (4.76–5.48)	<0.0001	
Model 3	Reference	1.09 (1.04–1.15)	4.13 (3.93–4.34)	4.66 (4.34–4.99)	<0.0001	
**Female**						
Event/Total	1239/11072	729/4184	313/592	258/370		
Incidence rate	10.59	17.10	74.72	126.28		
Crude model	Reference	1.61 (1.47–1.76)	7.04 (6.21–7.97)	11.93 (10.42–13.65)	<0.0001	
Model 1	Reference	1.43 (1.31–1.57)	6.48 (5.72–7.34)	9.47 (8.26–10.86)	<0.0001	
Model 2	Reference	1.33 (1.21–1.46)	5.72 (5.04–6.48)	7.67 (6.67–8.82)	<0.0001	
Model 3	Reference	1.19 (1.08–1.31)	5.45 (4.81–6.19)	6.50 (5.64–7.48)	<0.0001	

Model 1: adjusted for age, smoking habits, alcohol consumption, physical activities, education, family history of diabetes, antihypertensives, and lipid-lowering drugs.

Model 2: further adjusted for *log (*TG/HDL-C) (continuous), blood pressure categories (non-hypertension, hypertension grade I, grade II, grade III), and eGFR (<90, >90 ml/min/1.73m^2).

Model 3: Model 2+BMI (continuous).

eTable 26. Association between co-exposure to IFG and hsCRP≥2 mg/L and incident type 2 diabetes across age subgroups (<60, ≥60 years)

	**Combination of IFG or not and hsCRP, HRs (95% CIs)**				***P*-trend**	***P-INTm***
	**hsCRP <2 mg/L & non-IFG**	**hsCRP ≥2 mg/L & non-IFG**	**hsCRP <2 mg/L & IFG**	**hsCRP ≥2 mg/L & IFG**		
**<60 years**						<0.0001
Event/Total	6071/46291	2367/13907	1937/4238	838/1497		
Incidence rate	12.98	17.30	62.69	87.41		
Crude model	Reference	1.33 (1.27–1.39)	4.82 (4.58–5.07)	6.67 (6.21–7.18)	<0.0001	
Model 1	Reference	1.33 (1.27–1.40)	4.67 (4.44–4.92)	6.48 (6.03–6.97)	<0.0001	
Model 2	Reference	1.27 (1.21–1.33)	4.30 (4.08–4.53)	5.74 (5.33–6.17)	<0.0001	
Model 3	Reference	1.18 (1.12–1.24)	4.14 (3.93–4.36)	5.14 (4.78–5.54)	<0.0001	
P-INTm: IFG status (yes or not) * hsCRP subgroup (<2, ≥2) = 0.2631; IFG status (yes or not) * *log*hsCRP=0.1712.						
**≥60 years**						
Event/Total	1276/9560	851/5364	517/971	358/647		
Incidence rate	14.69	17.48	88.19	98.46		
Crude model	Reference	1.19(1.09–1.30)	5.71(5.15–6.33)	6.37 (5.67–7.17)	<0.0001	
Model 1	Reference	1.18(1.08–1.29)	5.71(5.16–6.33)	6.36 (5.65–7.16)	<0.0001	
Model 2	Reference	1.15(1.05–1.25)	5.29(4.77–5.87)	5.74 (5.09–6.47)	<0.0001	
Model 3	Reference	1.09(1.00–1.19)	5.30(4.78–5.88)	5.33 (4.73–6.01)	<0.0001	
P-INTm: IFG status (yes or not) * hsCRP subgroup (<2, ≥2) = 0.3048; IFG status (yes or not) * *log*hsCRP=0.1663.						

Model 1: adjusted for sex, smoking habits, alcohol consumption, physical activities, education, family history of diabetes, antihypertensives, and lipid-lowering drugs.

Model 2: further adjusted for *log (*TG/HDL-C) (continuous), blood pressure categories (non-hypertension, hypertension grade I, grade II, grade III), and eGFR (<90,>90 ml/min/1.73m^2).

Model 3: Model 2+BMI (continuous).

eTable 27. Additive effect of IFG and hsCRP≥2 mg/L on type 2 diabetes risks across sex and age subgroups

**Main effects – hazard ratios**	**Male**	**Female**	**Age <60 years**	**Age ≥60 years**
IFG	4.13 (3.93–4.34)	5.37 (4.78–6.03)	4.14 (3.93–4.36)	5.30 (4.78–5.88)
hsCRP≥2 mg/L	1.09 (1.04–1.15)	1.17 (1.06–1.30)	1.18 (1.12–1.24)	1.09 (1.00–1.19)
Joint effect	4.65 (4.34–4.99)	6.68 (5.71–7.81)	5.15 (4.78–5.54)	5.33 (4.73–6.01)
RERI	0.43 (0.08–0.78)	1.14 (0.04–2.29)	0.83 (0.43–1.22)	-0.07 (0.80–0.66)
AP	0.09 (0.02–0.16)	0.17 (0.03–0.31)	0.16 (0.09–0.23)	-0.01 (-0.15–0.13)
S	1.13 (1.03–1.25)	1.25 (1.02–1.53)	1.25 (1.13–1.38)	0.99 (0.83–1.17)
**Attributable proportion, %**				
IFG	85.75	76.94	75.66	99.31
hsCRP≥2 mg/L	2.47	2.99	4.34	2.08
Joint effect	11.78	20.07	20.0	-1.61

All models were adjusted for sex, age, smoking habits, alcohol consumption, physical activities, education, family history of diabetes, antihypertensives, and lipid-lowering drugs, *log (*TG/HDL-C) (continuous), blood pressure categories (non-hypertension, hypertension grade I, grade II, grade III), eGFR (≥90, 60-90, 30-60, <30 ml/min/1.73m^2), and BMI (continuous).

Abbreviations: RERI, relative excess risk due to interaction; AP, attributable proportion due to interaction; S, the synergy index; others are as eTable 1.

eTable 28. Additive effect of IFG and hsCRP≥2 mg/L on type 2 diabetes risks across combined sex-age subgroups

**Main effects – hazard ratios**	**<60 years males**	**<60 years females**	**>60 years males**	**≥60 years females**
IFG	3.95 (3.74–4.18)	5.30 (4.64–6.07)	5.13 (4.59–5.73)	6.54 (4.91–8.70)
hsCRP≥2 mg/L	1.15 (1.09–1.22)	1.26 (1.14–1.39)	1.09 (0.99–1.21)	1.16 (0.94–1.43)
Joint effect	4.73 (4.35–5.15)	6.91 (5.91–8.07)	5.04 (4.41–5.76)	7.28 (5.42–9.76)
RERI	0.63 (0.21–1.05)	1.35 (0.18–2.51)	-0.18 (-0.95–0.59)	0.58 (-1.81–2.97)
AP	0.13 (0.05–0.21)	0.20 (0.05–0.34)	-0.04 (-0.19–0.12)	0.08 (-0.23–0.39)
S	1.20 (1.07–1.35)	1.30 (1.04–1.61)	0.96 (0.79–1.15)	1.10 (0.74–1.64)
**Attributable proportion, %**				
IFG	79.09	72.76	102.23	88.21
hsCRP≥2 mg/L	4.02	4.40	2.23	2.55
Joint effect	16.89	22.84	-4.46	9.24

All models were adjusted for smoking habits, alcohol consumption, physical activities, education, family history of diabetes, antihypertensives, and lipid-lowering drugs, *log (*TG/HDL-C) (continuous), blood pressure categories (non-hypertension, hypertension grade I, grade II, grade III), eGFR (≥90, 60-90, 30-60, <30 ml/min/1.73m^2), and BMI (continuous).

Abbreviations: RERI, relative excess risk due to interaction; AP, attributable proportion due to interaction; S, the synergy index; others are as eTable 1.

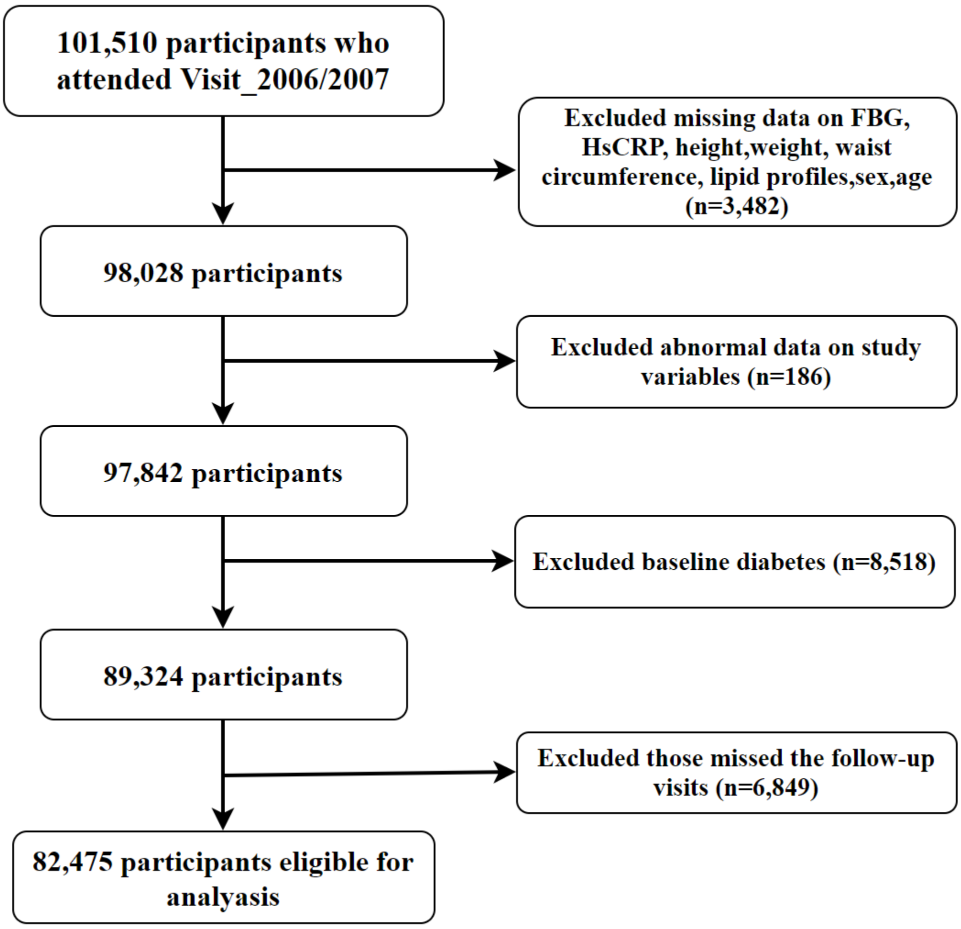

eFig 1. Flowchart of the study participants

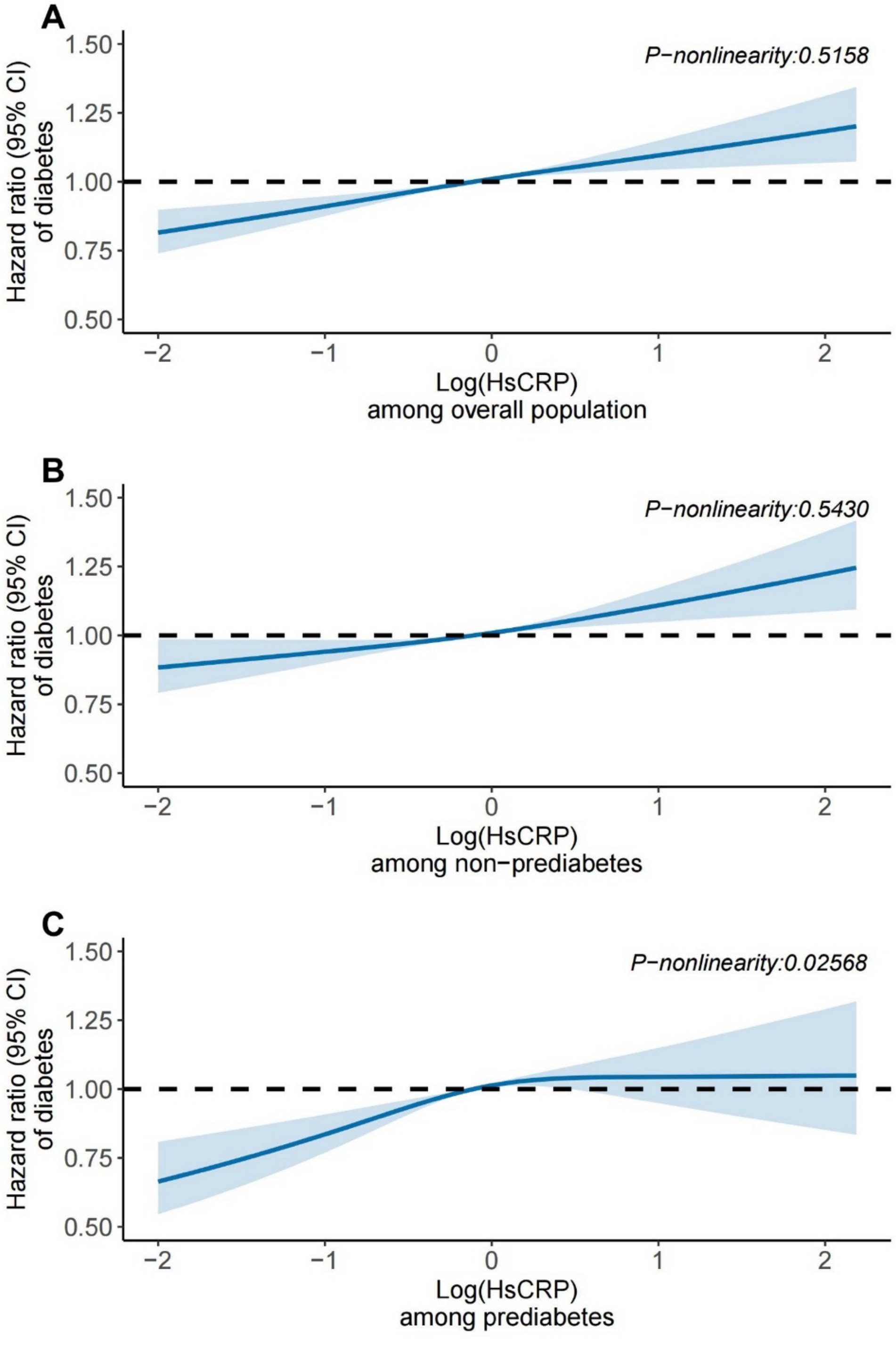

eFig 2. Dose–dependent association between log-transformed hsCRP and incident type 2 diabetes among the overall population (A), non-prediabetes (B), and prediabetes (C)

Multivariable-adjusted hazard ratios (solid lines) and 95% confidence intervals (shaded areas) were estimated using restricted cubic spline models with knots at the **10**th, 50th, and 9**0**th percentiles of log(hsCRP). The vertical dashed line represents log(hsCRP) = 0 (hsCRP = 1 mg/L). In the overall population and the non-prediabetes group, diabetes risk increased approximately linearly with higher log(hsCRP). In the prediabetes group, risk increased when hsCRP exceeded 1 mg/L and then plateaued.
